# The Yin and Yang of ACE/ACE2 Pathways: The Rationale for the Use of Renin-Angiotensin System Inhibitors in COVID-19 Patients

**DOI:** 10.3390/cells9071704

**Published:** 2020-07-16

**Authors:** Loris Zamai

**Affiliations:** 1 Department of Biomolecular Sciences, University of Urbino “Carlo Bo”, 61032 Urbino, Italy; loris.zamai@uniurb.it; Tel.: +39-0722-304319; 2 INFN-Gran Sasso National Laboratory, Assergi, 67100 L’Aquila, Italy

**Keywords:** severe acute respiratory syndrome coronavirus-2, eosinophil, asthma, IL-10, lung fibrosis, hypoxia, hypertension, cardiac dysfunction, renin-angiotensin system, Mas receptor

## Abstract

The article describes the rationale for inhibition of the renin-angiotensin system (RAS) pathways as specific targets in patients infected by SARS-CoV-2 in order to prevent positive feedback-loop mechanisms. Based purely on experimental studies in which RAS pathway inhibitors were administered in vivo to humans/rodents, a reasonable hypothesis of using inhibitors that block both ACE and ACE2 zinc metalloproteases and their downstream pathways in COVID-19 patients will be proposed. In particular, metal (zinc) chelators and renin inhibitors may work alone or in combination to inhibit the positive feedback loops (initially triggered by SARS-CoV-2 and subsequently sustained by hypoxia independently on viral trigger) as both arms of renin-angiotensin system are upregulated, leading to critical, advanced and untreatable stages of the disease.

## 1. Introduction

The novel severe acute respiratory syndrome coronavirus 2 (SARS-CoV-2) infection is the cause of the COVID-19 pandemic, for which there is currently no specific pharmacotherapy. In the intensive care unit, depending on the severity of the COVID-19 infection, patients might receive supportive care such as oxygen supply, interferons, glucocorticoids, anti-virals, chloroquine, and macrolides given as deemed appropriate. The SARS-CoV-2 infection produces either non-severe or severe symptoms and, in the latter case, it may precipitate into severe acute respiratory syndrome (SARS) and ultimately death [1,2,3,4,5,6,7,8,9]. Aging and different comorbidities are associated with patients’ critical status. Most patients display lymphopaenia and eosinopaenia, and critical ones show extremely low levels of eosinophils, suggesting that eosinopaenia may be a potential diagnostic biomarker [1]. Most serious cases of SARS are closely associated with cardiac dysfunction/injury, respiratory distress/illness, coagulopathy, hypoproteinaemia, acidosis, hypoxia and a cytokine storm mainly involving IL-1β, IL-6, IL-8, IL-10 and TNFα [1,2,3,4,5,6,7,8,9]. For instance, hypertension, an age-related disease, is the most common comorbidity (15–31% up to 24–58% in severe disease cases according to clinical reports), while only five cases of asthma (0.9%) were identified in one report of 548 infected patients in Wuhan [6], but no other asthmatic or allergic patients were described in other reports [1,2,3,4,5,7,8,9], considering that the overall prevalence of asthma is estimated to be 6.4% in Wuhan [6] and ~ 4% in China [10]. Viral infections usually increase the risk of allergic disease exacerbation [1]; however, the reported data seem to suggest that, in this case, the opposite is true. Indeed, in more than 72.000 patient cohorts examined, the number of patients defined as asthmatic was low (5), which suggests that asthmatics might be protected from virus-induced SARS, whereas pre-existing hypertensive disease and/or pre-existing antihypertensive treatments may represent a risk factor for increased virus infection, considering that the overall prevalence of hypertension in Chinese adult population ≥18 years of age is estimated to be ~ 23% [11]. Therefore, according to the clinical picture of infected patients, Th2-mediated allergic diseases (usually with high eosinophil counts) may play a protective role against SARS (usually with low eosinophil counts), while yet unknown mechanisms related to hypertensive conditions may exacerbate symptoms. To complete the picture, COVID-19 hospitalized patients suffering from chronic renal disease were usually low (0-3%) [1,2,3,4,5,6,7,8,9] if compared with the prevalence of the disease in China (10.2-11.3%) [12], suggesting that chronic kidney disease patients might be protected from COVID-19 as for asthma patients.

## 2. ACE2-mediated SARS-CoV and SARS-CoV-2 Infections

Angiotensin-converting enzyme 2 (ACE2) was identified as a receptor for the spike (S) protein of SARS-CoV, finally facilitating viral entry into target cells [13]. ACE2 is abundantly expressed in airway epithelial cells and vascular endothelial cells, and it is believed to play a crucial role in the mechanism of acute lung injury induced by SARS-CoV [14]. The ability of spike-Fc protein treatment (3h) to downregulate ACE2 protein expression has been shown in an in vitro system (cell lines) and also in vivo in lung cells of mice [14,15], suggesting that ACE2 pathway may be down-modulated during infection. However, ACE2 is constitutively expressed and released from the apical cell surface of human airway epithelia into airway surface liquid [16]. Of note, its surface down-modulation upon spike protein challenge has been shown to be due to ACE2 shedding mediated by activation of extracellular ADAM17/TACE metalloprotease, which concomitantly induces shedding/production of TNFα [17,18]. Interestingly, ACE2 shedding is enhanced not only by binding with spike protein [17,18], but also by IL-1β and TNFα inflammatory cytokines [16], cytokines that are secreted at relatively high concentration in COVID-19 patients [2]. Moreover, soluble (s)ACE2 (induced or not by virus binding) released from human airway epithelia has been demonstrated to retain both its enzymatic activity and its binding ability for spike viral protein, finally reducing spike protein-mediated viral entry into target cells [16,17]. Therefore, the interaction of ACE2 with spike protein of SARS-CoV induces a cellular “protective” ACE2 shedding feedback response that initially limits viral entry. Nevertheless, ADAM17/TACE-mediated ACE2 shedding or ACE2 enzymatic activity have been shown to intriguingly correlate positively with viral infection and disease complications [17,19,20]. In contrast, HNL63-CoV, which similarly binds to ACE2 through its spike protein, infects ACE2-bearing cells and mainly induces the common cold, leading to neither ACE2 shedding nor SARS [17]. Moreover, catalytically inactive forms of sACE2 can potently inhibit SARS-CoV infection [19,21], suggesting that events downstream of ACE2 shedding and/or its enzymatic activity may indirectly and subsequently favour viral infection and/or disease complications. To this regard, sACE2 was also associated with myocardial pathological conditions [22] and cardiovascular complications including hypotension (known to enhance both renin and angiotensin I, the substrate of ACE/ACE2) and tachycardia were common in SARS-CoV patients [23]. Since spike protein has been shown to not inhibit ACE2 enzymatic activity that is retained by sACE2-spike protein complex [16,17,24] and, in general, sACE2 maintains its enzymatic activity, we cannot consider its higher circulating expression a mere disease biomarker. Indeed, ACE2 shedding might repress its local function but it certainly enhances its circulating/systemic activity. Interestingly, a recent integrative bioinformatics analysis shows that the gene expression of ACE2 in human bronchial cells infected with SARS-CoV is dramatically increased 24h after infection and remained at a high level for at least 2 days, suggesting that ACE2 may be involved in a positive feedback loop post-infection [25]. In the same report, it has been shown that ACE2 expression level in bronchial epithelium obtained by brushing from asthmatic and normal subjects was similar, suggesting that respiratory epithelial cells of healthy subjects and asthmatic patients have similar ability to bind to SARS-CoV-2 through ACE2. Of note, ACE2 was also identified as the receptor for the novel spike protein of SARS-CoV-2 [13]. Although the role of ACE2 in the pathogenesis of SARS-CoV-2 and in inducing lung injury is still unknown, ACE2 behaves similarly to original SARS-CoV [13] (Box 1).

Box 1 **The formation of spike-ACE2 protein complexes may initiate ACE2 systemic upregulation and may impair autologous antibody generation/recognition of anti-S1-Receptor Binding Domain.**

The ACE2 interacts with the spike (S) protein, thereby serves as an entry receptor for SARS-CoVs, and this event is likely followed by conformational changes, cleavages and fusion of both spike viral and ACE2 proteins at the level of infected host cell plasma-membrane. The SARS coronavirus surface spike glycoproteins consist of trimers formed by two spike, capsid-distal S1 and –proximal S2, protein regions, located on the outer envelope of the virion. Spike proteins are clove-shaped trimers with three S1 heads and a trimeric S2 stalk that can bind the cellular ACE2 receptor when one S1 (possessing the receptor-binding domain) in the trimer adopts an “up” conformation (see Figure later in the Section 4). The subsequent binding of ACE2 receptor to the spike glycoprotein triggers both the ADAM17-mediated ACE2 shedding and dissociation of S1 fragments by exogenous (furin-related or other) proteases from the spike trimer, which leads on one hand to one S1-ACE2 complex and two S1 free fragments, and on the other hand to fusion of S2 viral trimers and cellular membrane structures, finally producing cell infection. The molecular mechanism model for SARS-CoV recognition and infection was well described by Song and collaborators [26] and a similar molecular interactions between SARS-CoV-2 and ACE2 has been recently hypothesized [27,28]. To complete the picture, a recent structural work reveals that the full-length human ACE2 is assembled as a dimer associated or not with amino acid transporter B^0^AT1, which sandwich ACE2 [28]. Binding of the spike protein trimer onto the ACE2 dimer suggests simultaneous binding of two spike protein trimers to an ACE2 dimer [28]. Each monomer of the ACE2 homodimer is composed of a membrane-proximal collectrin-like domain mediating homodimerization and necessary to position the molecule to the cell membrane, and of a membrane-distal ACE2 peptidase domain responsible for ACE2 substrate cleavage. Collectrin-like domain consists of an extracellular ferredoxin-like fold domain, also called neck domain (residues 616 to 726), a single transmembrane helix and an intracellular segment. The ACE2 cleavage site mediated by ADAM17/TACE has been predicted between amino acids 716 and 741 [16], which corresponds to neck-transmembrane boundary of ACE2 collectrin-like domain. It is tempting to speculate that, after ACE2 shedding, S2 viral trimers may fuse with the residual ACE2 collectrin-like fragments (complexed or not with B^0^AT1 transporters) which are eventually exposed on the cell surface membranes. A model for SARS-CoV’s internalization by target cells that was already proposed several years ago [29]. Then, when the virus is inside the cell, a second proteolytic cleavage mediated by endosomal proteases (such as TMPRSS2) in the S2 region of the spike-ACE2 fusion protein might be necessary for intracellular virus “release” and active infection.

Several S1-sACE2 complexes, free S1 fragments or sACE2 are likely released in bloodstream of COVID-19 infected patients by spike and ACE2 receptor cleavages. As already mentioned, circulating sACE2 protein shedding independent on virus contact has been also described either spontaneously when ACE2 transcription is upregulated or upon cytokine activation; it is therefore likely that in the bloodstreams ACE2 proteins are available to bind to both free S1 fragments and SARS-CoV-2 particles, finally leading to more and more S1-sACE2 and SARS-CoV-2-sACE2 complexes bearing enzymatic active sACE2 (see Figure later in the Section 4). Of note, SARS-CoV-2-sACE2 complexes, sequestrating sACE2, can drive part of the sACE2 systemic activity and concentrate it locally where the organs possess cells expressing ACE2 on their surface membrane, i.e., following the viral tropism (see Figure later in the Section 4). Indeed, although the viral load in sputum of adult SARS-CoV patients was usually higher than 10^4^ copies/mL, reports indicate that plasma viral RNA concentrations are low, usually lower than 190 copies/mL and lymphocytes have much higher RNA-copy concentrations of SARS-CoV RNA than plasma [30]. Very low RNA concentrations with no difference between patients with mild or severe symptoms were detected in plasma from COVID-19 patients [30]. The viral load in nasal swabs of asymptomatic and symptomatic COVID-19-positive subjects was also not significantly different [31]. Of interest, SARS-CoV infected pediatric patients have more than double the amount of plasma RNA copies/mL when compared to adult patients [30], suggesting a different viral tropism between adults and children (different cellular ACE2 expression possibly leading to Kawasaki disease). Notably, free S1 fragments may have a decoy function for autologous/exogenous anti-S1-receptor binding domain (RBD) antibodies. On the other hand, sACE2-S1 complexes masking S1-RBD might also impair either generation of autologous high affinity antibodies against S1-RBD or recognition/inactivation of S1-RBD on SARS-CoV-2 by autologous/exogenous antibodies. Therefore, antibodies that do not compete with ACE2 for the binding to S1-RBD might be more effective in neutralizing SARS-CoV-2 [32,33], knowing that the number of spike proteins per virion is estimated to be around 70 and that every spike protein bears three S1 receptor binding domains, which are masked to autologous antibody recognition when in a “down” conformation. These highly organized molecular features clearly demonstrate that the coronaviruses are armed with sophisticated infection machinery able to evade immune responses induced or not by vaccination. Nevertheless, passive immunotherapy by means of convalescent plasma from COVID-19 recovered donors is a promising option for prevention and treatment of COVID-19 [32,33,34,35,36,37].

## 3. Pathological Effects of ACE2 Pathway Upregulation

**Figure 1:**
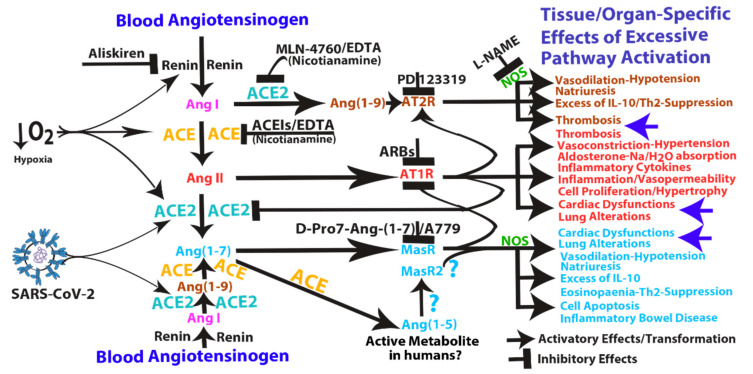
Interplay of regulation between the two arms of the renin-angiotensin system and pathophysiological consequences of excessive systemic activation of different RAS pathways. Reciprocal (ACE/ACE2) pathway inhibition, RAS inhibitor sites of action and influence of hypoxia/SARS-CoV-2 on the RAS are indicated. (for reference, see the text).

**Figure 2:**
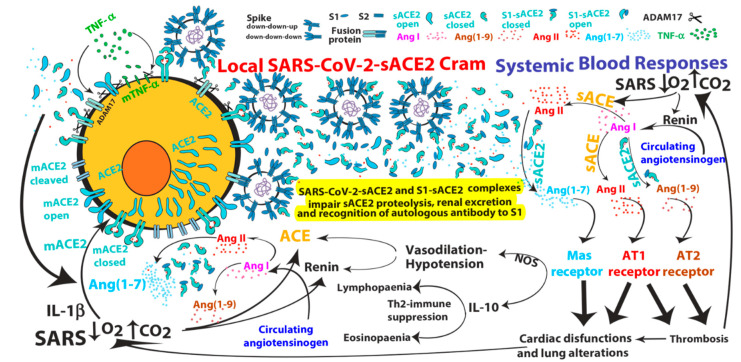
SARS-CoV-2 induced positive feedback loops mediated by RAS activation and pathophysiological consequences of the systemic excess of circulating ACE2 enzyme. (for reference, see the text).

ACE and ACE2 are two zinc metalloproteases involved in the biogenesis of the components of renin-angiotensin system (RAS). ACE2 processes angiotensin (Ang) I and II into Ang (1–9) and Ang (1–7), respectively, and it also has other known peptide targets, such as des-Arg(9)-bradykinin, which mediates B1 receptor activation. Ang (1–7) and Ang (1–9) peptides, opposing the effects of ACE/Ang II/Ang II type 1 receptor (AT1R) pathway, are known to mediate vasodilatative (hypotension), antiproliferative and apoptotic effects through Mas and AT2 receptors, respectively, both involving downstream nitric oxide synthase (NOS) pathway activation [38,39,40,41,42,43,44,45,46]. Physiology teaches us that there is a range of normality for every biological parameter, below (defect) and above (excess) of which there is a dysfunction. This is likely to be true for ACE as well as for ACE2. It is well known that excessive Ang II/AT1R pathway activation induces vasoconstriction, aldosterone, inflammation, excessive cell proliferation, thrombosis, cardiac dysfunction and lung alterations (see Figure 1). Moreover, most of the experiments show that increased ACE2 activity leads to beneficial effects; however, the experiments were usually performed using models in which its “antagonist“ (ACE) pathway was upregulated or ACE2 itself was downregulated, therefore balancing an unbalanced situation. What does it happen in models in which the opposite occurs?

(s)ACE2 or Ang (1–7) upregulation have been associated to some pathological conditions such as inflammation of the renal and gastrointestinal tract, cardiac dysfunction, human cirrhosis and lung injury/fibrosis. Tissue/organ specific effects of excessive pathway activation that in some case resemble those produced by excessive Ang II/AT1R pathway activation (see Figure 1). For example, systemic infusion of Ang (1–7) into mice had renal proinflammatory properties mediated by Mas receptor (MasR) activation [47]. Moreover, Ang (1–7) infusion was associated with increases in blood pressure, cardiac hypertrophy and fibrosis in rats with subtotal nephrectomy [48]. Similarly, elevated plasma sACE2 activity was associated both with greater severity of myocardial dysfunction and with an independent prediction of adverse clinical events [22,49]. Transgenic mice with increased cardiac ACE2 expression suffered from lethal ventricular arrhythmia (heart block, ventricular tachycardia and terminal ventricular fibrillation) consequent upon downregulation of connexins involved in gap junction formation [50]. In another model, ACE2 transgenic mice suffered from cardiac fibrosis with concomitant deficits in ejection fraction and fractional shortening [51]. Interestingly, in a rat model of myocardial infarction following coronary artery ligation, there is evidence that C16/MLN-4760 (a specific ACE2 inhibitor, see later) administration inhibits fibrosis and hypertrophy of non-infarcted myocardium and increases diastolic relaxation, raising the possibility that ACE2 activity may have some adverse effects on post-myocardial infarction heart [52]. The risk factors for cardiovascular disease include alterations in platelet function and coagulation with an increased risk of thrombosis. Ang II and hypercholesterolemia are known to participate in microvascular thrombosis and enhanced thrombus formation in the microvasculature may contribute to microinfarctions. To this end, in a rat model in which AT1R activation produce baseline thrombosis by platelet aggregation, Ang (1–9), known to bind AT2R [42], has been shown to enhance the thrombotic process [53]. Moreover, in a mouse model, AT2R activation (inhibited by AT2R antagonist, PD12319) mediated the onset of arteriolar microvascular thrombosis following chronic Ang II infusion [54], indicating both the recruitment of the AT2R pathway downstream of AT1R activation and its involvement in arteriolar thrombosis. Of interest, disseminated intravascular coagulation is associated with hypoproteinaemia and deficit of coagulation and anticoagulation proteins, which can originate by their renal loss (and consequent proteinuria) and/or by their increased (pathological) consumption and/or by their reduced hepatic synthesis. To this end, in healthy livers, ACE2 is limited to perivenular hepatocytes and endothelial cells; instead, in human hepatitis C cirrhosis, ACE2 protein expression is widespread in the hepatic parenchyma [55]. Notably, human hepatocytes cultured in hypoxic conditions upregulated ACE2 protein expression [55] and ACE2 mRNA, protein and activity were increased in response to hypoxia and by IL-1β [56]. In line with these observations, in peripheral blood human CD34^+^ cells, MasR expression and ACE2 expression, activity and shedding (of sACE2 catalytically active forms) were increased under hypoxia [57]. Moreover, under hypoxic conditions human pulmonary artery smooth muscle cells upregulated both (arms of the RAS) ACE and ACE2 mRNA and protein expression, and ACE2 was subsequently downregulated by (ACE-derived) Ang II through an AT1R-mediated process [58]. Similarly, both ACE and ACE2 expression were increased in vascular endothelium and smooth muscle of human left ventricle from patients with ischaemic (local hypoxia) heart disease [59]. Concomitant upregulation of both arms of the RAS that suggests a tight link between ACE and ACE2 under hypoxic conditions. Indeed, several reports have shown a complex interplay of regulation between the two arms of the RAS independently on hypoxia (see Box 2). Interestingly, chronic hypoxia induced activation of ACE2/Ang (1–7)/MasR axis and suppression of ACE/Ang II/AT1 receptor axis in lungs of pulmonary hypertensive Ren-2 transgenic rats (constructed by inserting the mouse *Ren*-2 renin gene) but not in normotensive transgene-negative control rats [60], suggesting that the baseline renin activity in hypertensive rats may be crucial to determine the differential response to hypoxia and that renin inhibition might be useful to inhibit the ACE to ACE2 pathway shift under hypoxic conditions. Of interest, hypoxia alone or combined with hypercapnia has been shown to significantly increase both plasma renin expression or activity and plasma Ang II expression [61,62]. Moreover, hypercapnic acidosis (pH 6.8/6.9, a condition that could occur in SARS) induced in isolated rat lungs has been shown to induce a (compensatory) venular dilatation mediated by cyclooxygenase activation (inhibited by indomethacin) [63], an enzyme that has been shown to be induced downstream of Ang (1–7)/MasR pathway in isolated rat hearts (again inhibited by indomethacin) [64], suggesting the involvement of ACE2/Ang (1–7) pathway in mediating CO_2_-dependent lung venular vasodilation**.** Altogether these observations indicate that there is a high probability that hypoxia/hypercapnia, a condition that occurs in SARS patients, might upregulate the activity of both arms of the RAS by suppling high amounts of renin product, Ang I, to ACE and ACE2, which, together, can produce high amounts of Ang II, Ang (1–9), Ang (1–7) and its (ACE-produced) metabolite, Ang (1–5) (see Figure 1). Similarly to both Ang (1–7)/MasR and Ang (1–9)/AT2R pathways, Ang (1–5) has been shown to induce the secretion of atrial natriuretic peptide via MasR/NOS pathway in isolated perfused rat atria [65], indicating that Ang (1–5) is a heart active peptide (at least in Sprague-Dawley rats). Interestingly, in the plasma of patients with inflammatory bowel disease, Ang (1–7) concentrations and (s)ACE2 activity were higher compared to healthy subjects [66]. Moreover, Ang (1–7) alone can either activate the Bax/Caspase–dependent apoptotic pathway or upregulate NF-kB signaling in lung fibroblast cultures [67]. Moreover, in vivo administration of Ang (1–7) alone (in Wistar rats) promoted morphological lung alterations, extracellular matrix accumulation and inflammatory cytokine production (including TNF-α and IL-6), characteristics of lung inflammation in pulmonary fibrosis [67]. Thereby, a condition that deteriorates respiratory compliance, could lead to hypoxia/hypercapnia. Hypoxia, in turn, will induce ACE2 upregulation which possibly increases cardiac/lung detrimental effects mediated by Ang (1–7)/MasR pathway activation finally leading to further ACE2 cell membrane upregulation. A condition that in COVID-19 can increase the probability of both SARS-CoV-2 entry and ACE2 shedding/systemic activity, finally generating positive feedback loops that might sustain SARS independently of viral infection (see Figure 2). This hypothesis is supported by the observations that, in lung aspirates of acid- and/or spike-treated mice, Ang II and ACE2 are synergistically upregulated and cell surface downregulated (shed), respectively, suggesting their involvement in the increased lung microvascular permeability and pulmonary oedema [14,15]. Indeed, this condition might subsequently favour the diffusion, in neighbouring lung tissues and systemic circulation, of both (s)ACE2, Ang II and Ang (1–7), the Ang II-derived product of (s)ACE2 processing. As already proposed, enhanced ACE2 shedding may locally reduce ACE2 activity in lung, however, it likely increases ACE2 systemic activity and subsequent production of circulating Ang (1–7). Interestingly, Ang (1–7) has been also reported to promote eosinophil apoptosis in lungs and in bronco-alveolar lavage fluid (BALF) [40]. Moreover, Ang (1–7)/MasR axis inhibits allergic airway inflammation and eosinophil cell counts in the BALF of a murine model of asthma, indicating both that an impairment of ACE2 pathway may favour asthma and that ACE2 pathway activation can reduces asthma symptoms [68]. Moreover, a compound that mimics the Ang (1–7) actions has been shown to induce IL-10 upregulation via a MasR-dependent pathway in BALF [69] and IL-10 is thought to mediate anti-inflammatory effects of MasR pathway activation [70]. IL-10 secretion in mouse plasma was also induced by an AT2 receptor agonist and downstream nitric oxide signalling [71]. Increased IL-10 production (e.g., by T regulatory cells) is often associated with immune tolerance, which is the consequence of reduced number and function of specific immune compartments [72]. Indeed, IL-10 has been shown not only to suppress antigen-specific Th2-mediated immune responses including eosinophil expansion in allergic inflammation [72], but also to augment airway reactivity, suggesting that despite its potent anti-inflammatory activity, an excess of IL-10, as well as Ang (1–7), may contribute to the decline in pulmonary function [73]. Of note, IL-10 is one of the cytokines downstream ACE2 pathway [25] and is significantly upregulated in the most severe forms of COVID-19 [2,8,9], indicating an important correlation between ACE2/Ang (1–7) axis activation and IL-10 upregulation which might lead to eosinopaenia/lymphopaenia in COVID-19 patients.

Box 2 **The reciprocal feedback-loop mechanisms of both arms of the RAS.**

Similarly to hypoxic conditions, a strong correlation between the gene expression of ACE and that of ACE2 was also observed in human renal cortical biopsy specimen, suggesting a link between the two gene transcription, possibly related to the amount/excess of their substrates (Ang I and Ang II) and not exclusively to hypoxia [74]. A link that tends to maintain the balance of ACE/ACE2 ratio, but that may be disrupted by ACE inhibitors (ACEIs) or by Ang II type 1 receptor blockers (ARBs) which both have been shown to upregulate ACE2 mRNA expression in left ventricle of Lewis rats, possibly through two different mechanisms involving upregulation of Ang (1–7) or Ang II, respectively [75]. In this regard, in cardiac myocytes isolated from neonatal rats, Ang II significantly reduced ACE2 mRNA and its activity, effects blocked by ARBs, indicating that Ang II downregulates ACE2 expression/activity through an AT1R-dependent mechanism [76] (see Figure 1). Moreover, in both cardiac myocytes and rat aortic vascular smooth muscle cells, Ang (1–7) prevented the Ang II-mediated reduction in ACE2 mRNA, an effect blocked by a selective MasR antagonist, D-Ala7-Ang-(1–7) (also known as A779), indicating that Ang (1–7) downregulates Ang II/AT1R signalling through a MasR-dependent mechanism [76,77] (see Figure 1). Therefore, Ang (1–7) (as well as ACEIs and ARBs), preventing Ang II-mediated ACE2 downregulation, shift the Ang peptide balance in favour of Ang II metabolisation by ACE2, which, in turn, leads to further production of Ang (1–7), finally sustaining ACE2 transcription, membrane protein expression and eventually shedding and systemic activity. Altogether these observations indicate a complex interplay of regulation between the two arms of the RAS in which feedback mechanisms of reciprocal (ACE/ACE2) pathway inhibition are involved at different levels: Ang II/AT1 receptor mediates a negative feedback signal on the ACE2 expression/activity and Ang (1–7)/MasR mediated a negative feedback signal on the AT1 receptor activity (see Figure 1). These reciprocal inhibitions in some cases (e.g., hypoxia/SARS-CoV-2 infection) can give rise to positive feedback loops that markedly shift the balance between Ang II/AT1R and the antagonist Ang (1–7)/MasR pathway (see Figure 1). For example, under hypoxic conditions both arms of the RAS are upregulated and the presence of SARS-CoV-2 can affect Ang (1–7)/Ang II balance (which might be further influenced by ACEI/ARBs) by shifting it in favour of an increased ACE2 systemic activity, Ang (1–7) production and MasR activation. This, in turn, can lead to further ACE2 cell membrane expression (increasing the probability of viral entry), which, after ACE2 shedding by binding to spike-SARS-CoV-2, ultimately establish a positive feedback loop.

## 4. COVID-19 Can Induce RAS-mediated Positive Feedback Loops at Different Levels

Intriguingly, the binding affinity of ACE2 to SARS-CoV-2 binding domain has been reported to be either equal to or 10- to 20-fold higher than ACE2 binding to SARS-CoV [27,32]. The affinity of spike protein for ACE2 has been shown to correlate with the severity of disease [24]. Although SARS-CoV produces more severe respiratory symptoms than NL63-CoV does, both viral receptor binding domains bind to ACE2 with similar affinity [78], indicating that SARS development is not related to the strength of binding affinity and depends on other mechanisms. In contrast to SARS-CoV, NL63-CoV did not induce ADAM17/TACE-mediated both ACE2 shedding and TNF-α secretion [17], suggesting that increased cleavability of ACE2 receptor and possibly of S1-S2 boundary may be crucial for disease severity. Indeed, the spike glycoprotein of SARS-CoV-2 but not of SARS-CoV, contains a furin-like cleavage site at the S1-S2 boundary which indicates an increased cleavability [13]. Of note, TNF-α and IL-1β were shown to both be upregulated in SARS-CoV-2 [2] and induce viral-independent ACE2 shedding from epithelial airway cells [16]. Moreover, viral-independent ACE2 surface release from epithelial cells was not only inducible by cytokines (e.g., TNF-α and IL-1β) but also constitutively and spontaneously produced when ACE2 surface expression was upregulated [16], for example upon IL-1β stimulation [56]. This suggests that systemic release of proinflammatory cytokines such as TNF-α and IL-1β can mediate an increase of sACE2 and its systemic activity. Of note, activation of ADAM17/TACE metalloprotease was induced by SARS-CoV and necessary for efficient infection (and TNF-α secretion) [17]. Intriguingly, both SARS-CoV infection and concomitant TNF-α secretion were significantly attenuated not only by knock-down of ADAM17/TACE expression by siRNA but also by deletion of the ACE2 cytoplasmic tail which is responsible for ADAM17/TACE activation [17]. Altogether these observations suggest the possibility that SARS-CoV may induce a positive feedback loop leading to surface expression and shedding of both ACE2 and TNF-α. Indeed, upon spike protein binding to ACE2, downstream pathway activation can sustain positive feedback loops at different levels (Figure 2):

(1) SARS-CoV can induce IL-1β and TNF-α systemic secretion that can mediate viral-independent surface membrane ACE2 upregulation and shedding. Of note, ACE2 shedding, on one hand, protects from viral infection but, on the other hand, increases circulating/systemic active sACE2, leading to its downstream pathway activation.
(2) Hypoxia in combination or not with hypercapnia can upregulate the activity of both arms of the renin–angiotensin system by inducing renin, ACE and ACE2 synthesis, which can increase expression of Ang I, Ang II, Ang (1–7), Ang (1–9), Ang (1–5) and the inactive metabolite bradykinin (1–7), but also membrane bound ACE2, finally giving more chances to SARS-CoV-2 entry.
(3) ACE2 can induce vasodilatative hypotensive effects by Ang II catabolism. Hypotension can induce again renin and ACE upregulation finally providing further Ang II, as a ACE2 substrate for further Ang (1–7) production.
(4) Ang (1–7) antiproliferative and apoptotic effects, possibly in part through IL-10, may mediate eosinopaenia and lymphopaenia that, on one hand, reduce inflammatory responses but, on the other hand, impair immune system ability to counter virus infection, finally predisposing the organism to further infections. Ang (1–7) immunosuppressive activity, mediated or not by IL-10, may also support the reduced ability to generate an effective immunization to SARS-CoV-2 infection.
(5) Ang (1–7)/MasR pathway can sustain ACE2 synthesis even in the presence of elevated concentrations of Ang II (such as in hypoxia), by inhibiting Ang II/AT1R-mediated down-modulation of ACE2 activity (see Figure 1).
(6) Ang (1–7)/MasR pathway can produce cardiac dysfunction and lung alteration leading to systemic hypoxia, which, in turn, upregulates the activity of both arms of the RAS.
(7) Although the ACE2 catalytic efficiency is 400-fold lower with Ang I than with Ang II [79], high concentration of circulating ACE2 may be able to produce significant increase of Ang (1–9) that, by binding AT2 receptors, can produce arteriolar microvascular thrombosis and local hypoxic conditions finally inducing local upregulation both arms of the RAS.

Finally, ACE2-loaded SARS-CoV-2 virions and S1-ACE2 complexes may impair both sACE2 proteolytic degradation by blood proteases and its renal excretion, therefore blunting the removal of its systemic enzymatic activity and indicating that circulating viral particles are dangerous even when they are not able to entry into the cells (see Figure 2). On the one hand, ACE2 hypertranscription and consequent increase of membrane (m)ACE2 exposure induced by hypoxia/hypotension may facilitate SARS-CoV-2 entry and its lifecycle into mACE2 expressing cells. On the other hand, the release of ACE2 from the cell membranes and its subsequent activity in the bloodstream and in local (lung/cardiac) extracellular fluids are likely critical steps in contributing to systemic disease pathogenesis. Nevertheless, the same aetiological agent, SARS-CoV-2, can produce a variety of clinical syndromes, which involves several organs in relation to the subject’s predisposition. Although in the present work I have supposed that most of the COVID-19 symptoms might derive from Ang (1–7), Ang II and Ang (1–9) peptide upregulation, the involvement of B1 receptor pathway suppression mediated by ACE2 metabolisation of des-Arg(9)-bradykinin to inactive bradykinin (1–7) or other ACE2-downstream peptides such as apelins, casomorphins and dynorphins cannot be rule out.

Based on the above observations, in order to block the RAS-induced positive feedback loops, different pharmacological targets can be hypothesized and pursued alone or in combination. They will be discussed in the next section and Box 3.

Box 3 **Possible targets of therapeutic intervention to inhibit the RAS-induced positive feedback loops.**

Pharmacological inhibition of enzymes involved in both viral entry, such as TMPRSS2 and ADAM17, and the RAS function such as renin, ACE and ACE2 enzymatic activity or their downstream pathways are expected to stop positive feedback loops and their detrimental consequences.

*Inhibition of TMPRSS2 and ADAM17 metalloprotease activity.* Inhibition of the serine protease TMPRSS2 (necessary for SARS-CoV-2 entry) by a clinically proven protease inhibitor has been recently suggested by Hoffmann and colleagues [13] and inhibition of ADAM17 enzymatic activity has been already proposed about ten years ago by Haga and colleagues [20]. Indeed, inhibition of ADAM17-mediated ACE2 shedding is expected to increase membrane ACE2 expression and therefore the probability of viral entry; nevertheless, in the early phases of the disease, inhibition of ACE2 circulating activity might be sufficient to inhibit the systemic RAS pathway upregulation and the development of severe forms of COVID-19. It is, in fact, possible that maintenance/recovery of correct organismal immune responses, by preventing ACE2-mediated immune suppression, in concert with cellular adaptive immune responses mediated by apolipoprotein B mRNA editing enzyme, catalytic polypeptide-like (APOBEC) systems [80] may anyway work to induce both an effective “immunization” and the viral eradication.

*Inhibition of both arms of the RAS.* Among the inhibitors of the RAS pathways, different strategy can be pursued involving either ACE2 enzymatic activity or its upstream renin and ACE enzymatic activity or its downstream MasR pathway. Inhibition of ACE2, ACE and renin enzymatic activities and their involvement in SARS-CoVs will be extensively discussed in the next sections, instead a brief description of MasR inhibition will be presented in the present Box.

*Mas receptor pathway inhibition and side effects of using Mas receptor inhibitors.* A779 also known as D-Ala7-Ang-(1–7) and D-Pro7-Ang-(1–7) are two distinct MasR antagonists able to prevent Ang-(1–7)-mediated downstream activation in human cells. The existence of several MasR subtypes has been suggested based on the differential capacity of the two MasR blockers to fully inhibit some biological actions of Ang-(1–7) [and perhaps of Ang (1–5), see Figure 1] [39,70]; therefore, differently from ACE2 enzymatic inhibitors, MasR antagonists should be administered in combinations, in order to inhibit ACE2 hyperactivity. In human aortic smooth muscle cells, they have been shown to restore NADPH oxidase/NF-kB/iNOS inflammatory pathway induced by Ang II when it is inhibited by Ang (1–7) co-administration [81]. In mice studies a MasR blocker (A779) administered alone was not associated with systolic blood pressure alterations, and the hypotensive effect produced by rACE2 co-infused with Ang II was unaffected by A779 co-administration, indicating that the hypotensive activity of rACE2 mainly depended on Ang II degradation rather than on increase of Ang (1–7) and MasR activation [82]. In another report, spontaneously hypertensive rats (SHRs) that received A-779 alone for a total of two weeks did not significantly alter basal blood pressure and urinary protein excretion [83]. Moreover, in SHRs treated with A-779 in combination with Ang II, renal injury and interstitial infiltration of macrophages and T cells were surprisingly reduced as compared with SHRs treated with Ang II alone, suggesting a safe use of A-779 drug in in vivo infusions [83]. Another report showed that infusion of A-779 alone for 7 days did not produce a significant effect neither on blood pressure nor on heart rate in SHRs [84]. In a rat model of cardiac arrhythmia, administration of A-779 alone did not cause any significant alteration in the number of arrhythmic events, confirming that A-779 can be safely delivered to rodents in vivo. [85]. Although MasR antagonists has been shown to be safe in acute and chronic in vivo studies either with mice or rats, there are no data on administration in humans and the existence of different MasR subtypes in the vasculature require combinations of MasR antagonists to inhibit an excess of ACE2 activity as for example may occur in COVID-19 patients.

## 5. Mechanism of Action and Potential Risk of Using RAS Pathway Inhibitors Targeting ACE2

### 5.1. ACE2 (and ACE) Hyperactivity: Is It a Matter of (Free) Zinc?

ACE2 and ACE are two zinc metalloprotease that function differently despite their similarities. ACE2 is a monocarboxypeptidase (cleaves a C-terminal single amino acid from its substrate), whereas ACE is a dipeptidylpeptidase (releases a C-terminal dipeptide from its substrate) [86,87]. ACE2 has a substrate preference for hydrolysis between proline and a hydrophobic or basic C-terminal residue [79,86,87] and acts not only on the RAS and bradykinin peptides but also on the C-terminus of the apelin, casomorphin dynorphin peptides [79], possibly inhibiting Apelin-13 hypotensive action [44]. It is expressed in the heart, kidneys, liver, colon, small intestine, lung, brain and testes [39,44,45,46,76,77,87]. Among the tissues expressing ACE2 there are cardiomyocytes, endothelium, alveolar type II cells, ciliated airway cells, vascular smooth muscle cells and testicular Leydig cells [14,39,44,45,46,76,77,87,88,89]. ACE2 is present on the apical surface of epithelial cells of renal tubules, lung alveoli and vascular endothelia, in these last cells the production of Ang (1–7) induces autocrine activation of MasR axis leading to NOS-mediated vasodilation and consequently hypotension [14,44,45,46,86,87,88,89].

ACE2, as well as ACE, is a zinc metalloprotease that can be inhibited by zinc chelating EDTA and activated by high concentrations of chloride or fluoride anions and zinc cation [79,90]. Indeed, both enzymes have chloride- and zinc-binding sites [90]. It is conceivable that these sites are responsible for the anion and zinc activation effect in both homologous enzymes [90]. However, the presence of chloride ions increases Ang I cleavage by ACE and ACE2 (increasing both Ang II and Ang (1–9)) and decreases Ang II cleavage by ACE2, suggesting that chloride induces specific ACE2 conformational changes in its active site (see [44]). To this regard, in plasma samples of COVID–19 patients, chloride range is usually normal or slightly higher (99.6–107.0 mmol/L) than normal values (96.–106.0 mmol/L) [5]. Unfortunately, I was not able to find plasma zinc concentrations in COVID-19 patients. Zinc is involved in several cellular activities and its systemic concentration is tightly regulated by several factors. Of interest, the major transporter/reservoir of zinc in plasma is albumin [91], a protein that was detected at significantly low concentrations in SARS-CoV-2 as well as severe adult respiratory distress syndrome (ARDS, a COVID-19-like disease) (usually < 3.5 g/dL, normal range 3.5–5.4) and inflammatory bowel disease (IBD, a disease with increased ACE2 activity) [6,8,66,92] Since most of zinc in plasma is sequestered by albumin, the total amount of plasma zinc mostly depends on albumin concentration and even small changes in albumin’s capacity for zinc binding may have significant consequences. To this regard, reduced concentrations of plasma albumin is believed to increase plasma levels of free zinc, while reducing the total plasma zinc concentrations [91], a condition that was observed in ARDS patients [93,94]. Indeed, albumin can potentially deplete free bioavailable forms of zinc; vice versa, hypoalbuminaemia may increase free zinc levels in plasma. Therefore, some ARDS patients with low levels of both total zinc and albumin might have rather higher than lower free zinc levels [93]. The free zinc concentration, which describes the fraction of zinc that is loosely bound and easily exchangeable, represents the highly bioavailable (and toxic) part of plasma zinc but it is not clinically detected. To that end, zinc/albumin ratio might be a surrogate marker of free zinc levels. Indeed, only recently it was described a fluorescence-based method for determining the free zinc concentration in human serum samples [95]. With this method it was shown that free zinc concentration in sera from females was significantly lower than in males [95]. Moreover, free zinc concentration did correlate neither with total serum concentrations of zinc (or other metal ions such as iron) nor with age [95]. Notably, increased plasma zinc bioavailability consequent to reduced albumin levels induces zinc import into cells and likely increases the cellular functions associated to cellular zinc concentrations such as ACE and ACE2. Indeed, enzymatic activity of both enzymes seems significantly upregulated in ARDS patients as detected by analysis of Ang peptide concentrations in plasma (see Box later in ARDS topic). In addition, serum albumin levels in COVID-19 were usually very low (in severe forms is usually < 3.1 g/dL) [6,8], raising the possibility of a negative correlation between albumin concentration in plasma and increase of the RAS activity. In line with these hypotheses, both asthmatic/allergic disease patients and chronic kidney disease patients without a history of cardiovascular disease, which are protected from developing COVID-19, showed a significant decrease in both circulating zinc levels and ACE2 activity as compared to normal values [96,97,98,99]. Interestingly, chronic renal disease patients with low zinc levels had normal concentrations of albumin but higher urinary zinc excretion than healthy controls [97], suggesting that low ACE2 activity in chronic renal diseases (and protection from SARS) might derive from a higher free Zn^2+^ renal excretion. A similar mechanism mediated by a reduction of extracellular levels of free Zn^2+^ might be hypothesised for asthma patients, as well [98]. Unfortunately, I could not find information on plasma albumin concentrations for asthma patients; nevertheless, we might suppose that in these patients the albumin concentration is in normal range.

Intriguingly, inhalation exposure to ZnCl_2_/ZnO/hexachloroethane (the main ingredients in smoke bombs) induces both elevated plasma levels of zinc and ARDS with clinical pictures that strongly resemble those of COVID-19 [100,101,102]. Since both ACE2 and ACE enzymes are activated by high concentrations of chloride and zinc ions [79,90], it is possible to hypothesise an activation of both arms of the RAS in ARDS induced by smoke bombs as well as in COVID-19 patients. In addition, metal fume fever, a flu-like syndrome caused by inhalation of welding fumes (mainly containing zinc) can be a predictor for the development of respiratory symptoms and welders have an increased pneumonia and cardiovascular risk [103,104]. In this regard, metal fume fever has been shown to produce fever, fatigue, muscle ache, cough, dyspnea, and an increase of several cytokines including IL-1β, IL-6, IL-8 and TNF-α [103], symptoms and cytokines that have already been described for COVID-19 patients [2,8,9]. Notably, TNF-α was upregulated early while IL-6 and IL-8 increased later, suggesting a subsequent involvement of these latter cytokines in response to inhalation of welding fumes [103]. Moreover, elevated levels of zinc have been shown not only to stimulate pro-inflammatory cytokine secretion by monocytes, but also inhibit T cell functions (see [102]). Zinc supplementation that has also been proposed for COVID-19 treatment [105,106,107], is able to suppress allogeneic immune response (see [102]) and incubation of blood cells with welding fume particles resulted in a considerable increase not only of pro-inflammatory cytokines (including IL-1β, IL-6, IL-8, TNF-α) but also the tolerogenic cytokine IL-10 [104]. Similarly, in pulmonary arterial hypertension (PAH) as well as SARS-CoV, both arms of the inflammatory system (namely IL-1β, IL-2, IL-4, IL-6, IL-8, IL-10, IL-12 and TNF-α) have been shown to be upregulated [108,109,110], suggesting a common systemic pathway of activation in welding syndrome, ARDS, PAH and SARS-CoV infections. Finally, the correlations between zinc exposure (metal fume) and cytokine storm [103,104] suggests a possible link between free zinc increase and these type of diseases. More information on systemic zinc homeostasis and multiorgan effects of elevated levels of extracellular free zinc (such as in subjects inhaling ZnCl_2_ smoke) is available in Box 4.

Box 4 **Systemic zinc homeostasis and multiorgan effects of elevated levels of extracellular free zinc.**

Zinc absorption occurs throughout the gastrointestinal tract into enterocytes by zinc transporters of the Zrt- and Irt-like protein (ZIP) family that take up Zn^2+^, but also Cu^2+^ and Fe^2+^ ions, both of which can inhibit zinc absorption [111]. The bioavailability of zinc is affected by several factors among them the amount of zinc in food, its chemical form (from very low availability, e.g., zinc oxide, to comparatively high, e.g., zinc chloride salt) and its binding to other (intestinal absorbed or non-absorbed) food components [111]. For example, phytates (components present in foods like rice, cereals and corn) and folic acid can form insoluble complexes with zinc that decrease zinc absorption and increase fecal zinc excretion [111]. On the other hand, Zn^2+^ can form complexes with most natural amino acids (AA) that for example can come from digested proteins; each Zn^2+^ can be coordinated with the amine groups and carboxylate anions of two AAs (a molar ratio of 1:2). Interestingly, in an in vitro model, the formulation of either ZnCl_2_ salt or Zn-AA complexes were absorbed by enterocytes and zinc was released on the opposite side of the enterocyte membrane, thus mimicking the intestinal absorption and release into the blood stream [111]. It is therefore conceivable that zinc chelates of AA, as well as zinc salts, may participate in bioavailability and uptake of zinc. The uptake of zinc-AA complexes by AA transporters of human enterocytes suggests that B^0^AT1 neutral AA transporter associated with ACE2 [28] might also be involved in zinc-AA uptake (a similar mechanism might be supposable for insulin biosynthesis/secretion in pancreatic β cells [91]). Indeed, a recycling of zinc-AAs, possibly supplied by membrane bound ACE2 monocarboxypeptidase cleavage, for ACE2 synthesis could be also hypothesised.

Plasma zinc concentration is mainly regulated through a balance between intestinal absorption and renal excretion involving specific mechanisms sensitive to both dietary zinc availability and its cellular utilization. The majority of Zn^2+^ in blood binds to serum albumin, which represents the major zinc transporter/reservoir protein in plasma. On the other hand, labile-bound pool of Zn^2+^ labile complexes (e.g., of AA) and free Zn^2+^ are the biologically available forms of zinc for cellular internalization and/or enzymatic activities (0.1–1% of the total zinc in blood plasma under normal conditions). Free Zn^2+^ is subsequently accumulated by endothelial cells and zinc uptake and release by endothelial cells is the crucial step for redistribution of Zn^2+^ from plasma to tissues [91].

Zinc is considered a relatively nontoxic metal; however, several data report that excessive free extracellular zinc is toxic and induce apoptosis in different cell types [91,102]. Plasma excess of free Zn^2+^ is uptaken by ZIP transporters on the cell surface of each cells in contact with plasma (including immune cells, endothelial cells, neurons and cardiomyocytes), leading to increase of intracellular zinc that can modulate zinc associated cellular functions in both physiological and pathological conditions. Indeed, zinc is an important mediator/messenger involved in several cellular activities and the shift of zinc from plasma to cells may be significant for both normal physiological processes including energy metabolism, blood coagulation, and zinc signalling, and a range of disease states, including neurodegenerative and cardiovascular diseases, metabolic syndrome, inflammation, endothelial stress, diabetes and thrombosis [91]. For example, excess zinc intake has been shown to contribute to both amyloid beta-peptide plaque formation in Alzheimer’s disease and cardiac dysfunction, indicating that free zinc ion may be much more toxic than thought [91]. It has been reported that Alzheimer and other neurodegenerative disease patients show elevated levels not only of Zn^2+^ but also of other divalent metals such as Fe^2+^ and Cu^2+^ [112], suggesting a possible link between the increase metal ions and saturation of ZIP transporters. On the other hand, increased plasma ferritin levels (such as in COVID-19 [2,3,6]) might reduce free Fe^2+^ and its “competition” with Zn^2+^ for ZIP transporters, finally increasing cellular zinc uptake and functions. To this regard, EDTA metal chelator has been shown to protect from neural cell death and it was tested in neurodegenerative diseases [102]. Of interest, plasma albumin acts as a transport protein for both metal ions such as zinc and hydrophobic molecules such as free fatty acid (FFA) to allow their systemic distribution. Indeed, albumin is well beyond a mere carrier of metal ions and hydrophobic molecules, and by binding of these different molecules mediates their crosstalk and bioavailability [91]. To this regard, one particularly intriguing allosteric link exists between FFA and zinc binding to albumin [91]. When FFAs with ten or more carbon atoms bind to albumin causes the release of Zn^2+^, suggesting a possible link between dyslipidemia and toxic effects mediated by consequent increase of free zinc in plasma. For example, endothelial stress and activation and pro-inflammatory action of FFAs may be mediated by their effects on free zinc release from albumin and its consequent efflux from plasma. Different studies have suggested a role of albumin to inhibit interaction of free Zn^2+^ with amyloid beta-peptides and combat plaque formation in Alzheimer’s disease [91]. Zn^2+^ has also been shown to promote clot stability by binding to fibrinogen and inhibiting heparin activity [91]. An evidence that has suggested a possible positive correlation between increased levels of FFAs and thrombotic risk [91]. In addition, physiological conditions associated with elevated plasma FFAs, such as physical exercise, can also produce an increased plasma level of free zinc and its downstream physiological events (e.g., activation of the RAS). On the other hand, reduced concentrations of plasma albumin (such as IBD, ARDS and COVID-19) will induce elevated plasma levels of both free FFAs and bioavailable zinc. Intriguingly, ARDS is induced after inhalation exposure to ZnCl_2_/ZnO/hexachloroethane, the main ingredients in smoke bombs used for crowd dispersal [100,101,102]. To this regard, soldiers that breathed smoke-bomb fumes have been shown to quickly or slowly develope ARDS [100,101]. Interestingly, a patient who had the highest level of serum zinc rapidly developed ARDS that leads to death from multiorgan failure (including respiratory and hepatic failure) [100]. Instead, a slow progressive clinical course was associated with a significant increase of plasma zinc concentration that negatively correlated with pulmonary and liver function [100], finally leading to death for severe respiratory failure three to five weeks after inhalation [100,101]. At autopsy diffuse microvascular obliteration and marked endothelial cell injury was apparent [101]. Widespread occlusion of the pulmonary arteries may contribute to the development of acute pulmonary hypertension and extensive interstitial and intra-alveolar fibrosis was also observed [101]. Other soldiers wearing gas masks, immediately developed severe coughing and dyspnea, and slowly improved their lung function (only twelve months after exposure their lung function tests were nearly normal) [101]. All clinical conditions that strongly resemble those described in COVID-19 patients.

### 5.2. Safety and Efficacy Concerns of MLN4760 and Dx600 ACE2 Inhibitors.

It is known that the catalytic cleft of ACE2 consists of two peptidase subdomains: one membrane-distal and the other one membrane-proximal. Their weak interactions are consistent with the ability to transition from open to the closed ACE2 conformation [28,90]. Indeed, the two subdomains undergo a large hinge-bending motion in which membrane-proximal subdomain remains almost unchanged, while membrane-distal subdomain moves to close the distance between the two subdomains, mimicking the opening/closing movement of a clam shell [90]. ACE2 open conformation likely reflects free state of the enzyme available to catch substrates (or inhibitors), then, when the ACE2 receptor binds to a substrate, the membrane-distal subdomain closes around the substrate (or the inhibitor), finally performing the enzymatic activity [24,90]. Interestingly, in cryo–electron microscopy structures of full-length human ACE2, only the closed/substrate-bound conformation of ACE2 was observed in the spike-ACE2 complexes [28]. Since human ACE2 is assembled on cell surface as a homodimer [28], binding of the spike protein trimer onto ACE2 dimer suggests simultaneous binding of two spike protein trimers to substrate-bound conformer of ACE2 homodimer on plasma membrane. The spike binding sites on ACE2 homodimer are localized above the membrane-distal peptidase subdomain of each ACE2 monomer, nevertheless neither ACE2 shedding nor ACE2 binding to spike proteins have been shown to inhibit ACE2 enzymatic activity [16,17,24]. On the other hand, the S-protein-binding region of membrane-distal ACE2 subdomain is not significantly perturbed by the receptor conformational changes and maintains the ability to associate with soluble spike proteins independently on open/closed conformations [24]. Interestingly, an ACE2 specific inhibitor (MLN-4760) has been shown to induce the closed (inhibitor-bound) ACE2 structure [90] and to retain its inhibitory effects on sACE2 bound to spike proteins [24].

MLN4760 is a potent and selective human ACE2 inhibitor (IC50 = 0.44 nM against soluble human ACE2) whose synthesis produces a racemic mixture of two diastereomers that showed 75:25 ratio for Isomer A: Isomer B [113,114] and the purified isomer B is the isomer commercially available from Merck Millipore [114]. Testing MLN-4760 racemic mixture and its isomers, it was observed a concentration-dependent inhibition of recombinant human (rh)ACE or rhACE2 activities with all three inhibitors [114]. The isomer B was less selective (near minimal-maximal rhACE inhibition range 10^−6^M-10^−4^M) than the racemate or the isomer A (near minimal-maximal rhACE inhibition range 10^−6^M-10^−2^M) and less effective (near maximal rhACE2 inhibition at 10^−7^M) than the racemate or the isomer A (near maximal rhACE2 inhibition at 10^−8^ M) for rhACE2 versus rhACE [114]. Moreover, all three inhibitors exerted a significantly higher inhibitory activity against soluble than membrane-bound forms of (m)ACE2 (near maximal mACE2 inhibition at 10^−6^M), being the isomer B more selective and effective than the racemate or the isomer A for mACE2 vs. mACE expressed on the surface of mononuclear cells (or CD34 hematopoietic progenitors) [114]. A second ACE2 inhibitor, Dx600, produced similar results to those obtained with the isomer B [114]. Another study evaluated the inhibitory activity of both MLN-4760 and DX600 (either the linear conformational form or the disulfide bridged cyclic variant) inhibitors. In this report, the experiments were performed at pH 6.5 [115], a pH at which rhACE2 proteolysis operates at maximal activity [79] and a condition that resembles hypercapnic acidosis which may occur during SARS. MLN-4760 was still able to strongly and specifically inhibit rhACE2 activity (near maximal inhibition at 10^−8^ M), preventing rhACE2-driven Ang II degradation into Ang (1–7), whereas DX600 (either linear or cyclic variant) inhibits rhACE2 at relatively higher concentration (near maximal inhibition at 10^−6^ M–10^−7^ M, respectively) [115]. Altogether these data indicate that the racemate or the isomer A are more effective in inhibiting soluble forms of ACE2 than isomer B and Dx600. Therefore, these inhibitors at opportune (low) concentrations are expected to preferentially reduce systemic sACE2 activity, while preserving the ACE2 activity of (local) membrane-associated forms of ACE2, knowing that catalytic activity of circulating ACE2 was undetectable in human plasma of healthy subjects due to an endogenous ACE2 inhibitor [116].

Since MLN-4760 inhibitor promotes the closed ACE2 conformation [90] which is the preferential conformer for virus binding [28], MLN-4760 is expected to not prevent viral entry; nevertheless, its inhibitory function on ACE2 pathway might work on the positive feedback loops (above described) that ultimately favour ACE2 membrane expression and viral entry. A potential risk factor of inhibiting the Ang II metabolization into Ang 1–7 could be the increase of blood pressure. Although ACE2 pathway inhibition might lead to hypertensive effects, treatment with MLN-4760 for 4-5 weeks had no effect on blood pressure when administered 10 mg/kg/day in drinking water in wild type mice [117] nor in male (mRen2)27 transgenic hypertensive rats (administered 30 mg/kg/day subcutaneously via mini-osmotic pumps) [118], suggesting that hypertensive activity mediated by ACE2 inhibition is promptly balanced by compensatory mechanisms either in normal or hypertensive blood pressure conditions. In addition, injections of MLN4760 into the nucleus tractus solitarii has been shown to reduce the baroreceptor reflex in rats, suggesting a role for ACE2 in controlling a reflex bradycardia (see [39,44]). Finally, chronic inhibition of ACE2 with MLN-4760 led to increase of ACE, albuminuria and glomerular injury in streptozotocin-induced diabetic mice, indicating a possible adverse effect of the inhibitor in a diabetic background [119].

Extensive experiments have been also performed with DX600, a specific peptide ACE2 inhibitor that exhibited a mixed competitive and non-competitive type of inhibition [120]. Actually, several reports describing Dx600 inhibitor administration in mice suggest its safe use. Of interest, a report described its (safe) use alone (1 mg/kg per day) by nasal inhalation for 3 days in a mouse model of endotoxin-induced lung inflammation [121]; however, this inhibitor is less efficacious than MLN-4760 in inhibiting the soluble forms of human rACE2 [114,115]. Altogether these reports on administration of ACE2 inhibitors do not reveal significant adverse impacts or mortality in experimental animals, which suggests their safety in chronic administration. This was also confirmed in human clinical trials with MLN-4760 (clinical name: ORE1001, see later). Of interest, a report showed that on day 28 post-myocardial infarction, adult male Sprague-Dawley rats that had received MLN-4760 (also called C16) 25 mg/mL/day by daily intraperitoneal injection (as a solution of 42 mg/mL in distilled water) tended to have lower left ventricular pulse pressure, mean arterial pressures and left ventricular relaxation time constant-Tau compared to untreated group (see table 2 of the paper) [52], suggesting a possible protective role of ACE2 inhibition in post-myocardial infarction. Therefore, MLN-4760 might be helpful not only for COVID-19 but also in targeted therapies for pathologies correlated with an excessive increase of ACE2 activity that may involve heart, lung, liver, colon or other tissues/organs expressing ACE2 such as blood and endothelial cells. As an example, GL1001 (old name of MLN-4760) showed to produce an anti-inflammatory activity in a mouse model of colitis [122], highlighting the importance of the yin-yang balance of ACE/ACE2 pathways (“*in medio stat virtus*”).

### 5.3. Safety and Efficacy Concerns of Chelating Agents

Based on ACE2 mechanism of action, there are alternative ways to inhibit ACE2 (and ACE), for example concentration of cation/anion might also targets of intervention to inhibit the zinc metalloprotease ACE2 (and ACE). In this regard, EDTA, a cation chelator, has been shown to be able to inhibit ACE2 activity [79]. Of interest, EDTA binds with 10^6^- and 10^2^-fold higher affinity to Zn^2+^ than to Ca^2+^ and Fe^2+^, respectively [123], and in plasma, free Ca^2+^ concentration (1.05–1.30 mmol/L) is “only” about 10^3^-fold higher than free Zn^2+^ (0.1–2.0 µmol/L), while majority of iron in plasma is bound to transferrin. As a consequence, the iron concentration in plasma of healthy subjects is very low on the order of 10^−18^M [112]. Among the essential metal ions present in the organisms, the specific affinity of EDTA for systemic Zn^2+^ in physiologic conditions is highlighted by the evidence that prolonged treatment with CaNa_2_EDTA results in specific zinc depletion that is believed to mediate the teratogenic effects of the drug [123]. As already mentioned, zinc is an important mediator/messenger involved in several cellular activities and excess of free zinc has been shown to be toxic (see also Box 4); however, zinc deficiency has also detrimental effects on growth, neuronal development, and immunity, and in severe cases its consequences can be deadly [102]. For these reasons, treatments with CaNa_2_EDTA require a careful monitoring of the patient for the therapeutic effects as well as possible complications, so as to titrate the appropriate dosage.

CaNa_2_EDTA was approved by FDA in chelation therapy for lowering blood lead levels a long time ago (1953). Then, different (commercially available) iron chelating agents, expected to work in chelating zinc ion as well, were approved by FDA. Chelation therapy comprises intravenous or oral administration of chelating agents that remove metal ions such as zinc from the body. Cells synthesising high amounts of ACE or ACE2 needs of high amounts of bioavailable (free) zinc. Therefore, it is conceivable that plasma levels of free zinc may influence not only ACE and ACE2 activities but also their cellular synthesis. Indeed, both ACE and ACE2 synthesis might be particularly sensitive to reduction of free zinc levels in case of their upregulation. In addition, metal chelating agents, by limiting the availability of free zinc to cells, might have effects on both ACE2/ACE synthesis and conformation (when assembled on the plasma membrane). Indeed, the closed conformer of ACE2 homodimer that is the preferential conformation for virus binding [28], needs the presence of both zinc and substrate/inhibitor in the catalytic site [90], suggesting that zinc chelation (differently from MLN-4760) might also inhibit ACE2-mediated viral entry.

Intriguingly, chloroquine has been shown to specifically enhance zinc uptake and its accumulation/sequestration in the lysosomes [124], raising the possibility that it might work on COVID-19 patients by reducing zinc recycling and zinc functions. As already mentioned, free Zn^2+^ promotes clot stability by binding to fibrinogen (see Box 4 and [91]) and chloroquine has been shown to have an anti-thrombotic activity by inhibiting platelet activation [125]. To this regard, upon platelet activation the release of Zn^2+^ store from their secretory granules has been shown to participate to the pro-coagulant activity in platelet-dependent fibrin formation [126], suggesting that an elevated free Zn^2+^ concentration might occur and contribute to thrombotic predisposition in COVID-19 patients, a phenomenon possibly countered by chloroquine.

For all of the above reasons, cation chelating agents, administered alone or in combination with other therapies, might be effective to counter COVID-19 infection, in particular when, induced by hypoxia, both arms of the RAS are upregulated. A scenario that would deserve an investigation. However, some formulations of metal chelating agents carry a black box warning because they may cause serious and fatal renal toxicity and failure, hepatic toxicity and failure, gastrointestinal haemorrhage, arrhythmias, tetany, hypocalcaemia, hypotension, convulsions, respiratory arrest, and agranulocytosis that can lead to serious infections and death [123]. For the sake of completeness, besides renal toxicity linked to the route of drug excretion, EDTA has been shown to be effective in chronic renal artery diseases [123]. Finally, for its teratogenic effects, the drug is also contraindicated in pregnancy [123]. As a result, treatments with metal chelating agents require close patient monitoring, including laboratory tests of renal and hepatic function, and absolute neutrophil count should be monitored before and during treatment. Alternative ways of RAS pathway inhibition are also described in Box 5.

Box 5 **Alternative ways of RAS pathway inhibition.**

Of interest, a small cationic inhibitor of ACE2 (but not of ACE) has been detected in plasma samples [116]. The endogenous inhibitor might play a compensatory fine-control (within a threshold limit) for normal fluctuation of ACE2 protein in plasma and it has been hypothesized to be a basic AA or a small basic peptide able to compete with ACE2 substrates [116]. I have already mentioned that AA can chelate zinc forming labile zinc complexes of amino acids (see Box 4). It is therefore possible that a basic AA or a small basic peptide capable to specifically accommodate into the catalytic site of ACE2, but into that of ACE, could inhibit ACE2 activity. Indeed, despite the similarities, ACE and ACE2 possess different substrate specificity that depends on the smaller ACE2 binding pocket compared to that of ACE [90]. This specific aspect might determine the specificity for a specific basic amino acid or molecule that is able to accommodate into the catalytic pocket of ACE2 and bind to zinc. Among the possible endogenous ACE2 inhibitors, agmatine, decarboxylated arginine, has a chemical structure that resembles that of an ACE2 inhibitor, NAAE [127]. NAAE is a small molecule that had demonstrated an anti-SARS-CoV activity, by acting on both ACE2 catalytic activity and ACE2 binding domain for spike protein of SARS-CoV [127]. Unfortunately, NAAE has never been used in vivo. Indeed, NAAE is a weak ACE2 inhibitor, it is in fact more than a thousand-fold less potent than MLN-4760; however, if agmatine will be proven to have ACE2 inhibitory activity, it might be helpful to prevent the trigger of the positive feedback loops in the first mild phases of the disease. Indeed, it has an important role in down-regulating NO synthesis reducing NO overproduction by different mechanisms [128]. Of note, NOS pathway has been shown to be upregulated by both Ang (1–7)/MasR and Ang (1–9)/AT2 receptor pathways that are downstream ACE2 activity [38,39,41,43]. Moreover, agmatine has a regulated plasma concentration in the range of 20-80 ng/mL and the use of dietary agmatine has been shown to be safe and effective in reducing neuropathic pain [129]. Moreover, agmatine sulfate is regularly taken as a bodybuilding supplement.

Among natural metal-chelating agents, phytates and folic acid are two chelating agents from vegetables that might reduce zinc intestinal absorption and possibly its systemic concentration (see Box 4 and [111]). Similarly, nicotianamine that is extracted from plants (soybean) is a low-molecular weight metal chelator with high affinity for divalent metal cations. Nicotianamine has been shown to inhibit the activity of both zinc metalloproteases, ACE2 and ACE [130]. In addition, zeolites might also be effective in reducing free zinc availability. Zeolites are a group of aluminosilicate minerals with crystalline microporous structure that are originated from volcanic rocks. Their molecular structure generates cavities that allow high absorbency capacity for a wide range of charged elements such as water, heavy metals, cations and many toxins. Clinoptilolite is one of the zeolites that has been widely studied in veterinary and human medicine. The increased usage of clinoptilolite-based products in vivo has stimulated several investigations on its safety and its positive medical effects related to human health (see [131,132]).

Finally, soluble and catalytically inactive forms of ACE2 have been shown to be potent inhibitors of SARS-CoV infection products [19,21]. An approach that could be pursued (in combination with other therapies) to inhibit SARS-CoV-2 entry. Indeed, soluble forms of ACE2 are expected to protect from viral infection and a similar strategy using a recombinant form of human ACE2 has been proposed not only in COVID-19, but also in ARDS and PAH [108,133,134]. However, it is possible that catalytic active form of ACE2 might favour adverse effects in these specific pathological conditions. To this regard, a clinical trial using recombinant hACE2 protein has been recently started (ClinicalTrials.gov number, NCT04287686) in COVID-19 patients and pilot clinical trials of rhACE2 in ARDS and PAH started in 2017 and 2018, respectively [108,134]; unfortunately, no conclusive results are available yet.

## 6. Correlation of Pre-existing Circulating ACE2 Activity and Increased Potential to Develop Severe Forms of COVID-19

Severe symptoms of COVID-19 have been described to correlate with pre-existing hypertension, diabetes, age and male gender [1,2,3,4,5,6,7,8,9]. It is not still clear whether it depends on constitutive hypertensive conditions and/or on anti-hypertensive treatments or on other age-related conditions, considering that the prevalence of hypertension in Chinese adults is ~ 23% and that only about 41% take prescribed antihypertensive medications, being calcium channel blockers the most commonly used (~ 50%) in China [11]. To this regard, there is an interesting report describing sACE2 activity in plasma samples of Spanish healthy subjects and patients, in which a total of 2572 subjects from a multicenter study (NEFRONA project, 2009–2011) was studied [96]. The report shows that male and advanced age were identified as independent predictors of enhanced sACE2 activity [96]. Furthermore, subjects with hypertension, diabetes, dyslipidemia, or plaques also had significantly increased circulating ACE2 activity when compared with those without these pathologies [96]. Notably, hypertensive (the most frequent comorbidity with COVID-19) and diabetic patients are often treated with ACE inhibitors (ACEIs) and/or with ARBs, suggesting a possible positive correlation [135,136,137,138]. Interestingly, circulating ACE2 activity is significantly increased in subjects on therapy with ARBs or taking oral antidiabetic agents as compared with non-treated patients, while treatment with ACEIs and cholecalciferol had no significant influence on circulating ACE2 activity [96]. In line with this observations, losartan (an ARB), but not lisinopril (an ACEI), was able to upregulate ACE2 activity in left ventricle of Lewis rats [75]. Nevertheless, plasma concentration of Ang (1–7) was significantly increased with both treatments when compared to vehicle-treated rats and it was significantly higher in ACEI-treated than ARB-treated rats [75], suggesting that ACE inhibition is able to induce an activation of the ACE2/Ang (1–7)/MasR pathway. Although more evidence is needed in humans, the above observations suggest that ARBs should be precautionarily avoided to reduce possible ACE2-mediated viral consequences. Indeed, under hypoxic conditions, either an ACEI or an ARB have been shown to upregulate membrane ACE2 protein expression on human pulmonary artery smooth muscle cells [58]. ACEI and ARB have been shown to work by reducing the concentration of Ang II and by inhibiting its AT1-mediated ACE2 downregulation, respectively [58]. ACEIs and ARBs might therefore play a role in the upregulation of membrane ACE2 expression under hypoxic conditions such as COVID-19, knowing that the increase of membrane bound ACE2 (before its shedding) will also increase the probability of viral entry. Of note, ACEI and ARB antihypertensive medications are more commonly used in Europe/USA than in China. Nevertheless, recent reports show that the use of ACEI/ARB medications in patients with COVID-19 is safe and ACEI/ARB exposure was not associated with a higher risk of having severe forms of COVID-19 [6,139,140,141,142,143]. Moreover, some reports show that ACEI/ARB treated patients may even be protected from COVID-19 and ACEI/ARB exposure was associated with a lower risk of mortality compared to those on non-ACEI/ARB antihypertensive drugs [144,145,146,147,148]. Among these reports, one indicates that the risk of severe symptoms of COVID-19 was significantly decreased in patients who took ARB drugs (but not ACEIs) compared to patients who took no drugs [144]. Differently, another report suggests that the use of ACEIs (but not ARBs) reduced risk of death and/or critical disease [146], suggesting that some of these reports did not adjust for confounders and further analyses are need. Nevertheless, clinical trials of losartan as a treatment for COVID-19, are actually underway among patients who have not previously been treated with ARBs and/or ACEIs (NCT04312009 and NCT04311177). However, most of hypertensive/diabetic patients are ACEI- or ARB-“pretreated”, nevertheless they have an increased risk to develop SARS, therefore ACEIs and ARBs, if not detrimental, are not expected to face the disease in non-hypertensive patients. A possible beneficial effect of ACEIs in COVID-19 patients could indirectly come by reducing ACE2 substrate, Ang II, and finally limiting Ang (1–7) [but not Ang (1–9)] production and its (detrimental) effects; however, this is only a hypothetical possibility since, in this case, the ACE2/ACE pathway might be even more unbalanced.

To complete the picture, smokers and subjects on therapy with insulin tend to have an increased (although not significantly) circulating ACE2 activity when compared with control subjects [96]. On the other hand, increased ACE2 protein expression was reported in plasma and/or urine of physically active men after acute aerobic training or in renal cortices of spontaneous hypertensive, but not normotensive, rats after chronic aerobic training [149,150]. In addition, recent works reveal that asthma and other allergic diseases, which protect from developing COVID-19, were associated with significant reductions in levels of both zinc in plasma and ACE2 mRNA in airway cells [98,99]. On the other hand, as already mentioned, some patients with cardiovascular diseases (and inflammatory bowel disease) have an increased circulating ACE2 [22,49,151,152], which might explain the higher probability of elderly heart patients to develop COVID-19. Interestingly, in a report on atrial fibrillation, increased plasma ACE2 activity was significantly associated not only with cardiac dysfunction, increasing age, male gender and hypertension, but also with vascular disease [152]. Altogether the data suggest a strong correlation between circulating ACE2 activity and the predisposition to develop the most severe symptoms of COVID-19, suggesting that circulating sACE2 might be a predictive biomarker of SARS development. The surprising aspect is that circulating (differently from membrane bound) ACE2 is expected to protect from viral infection and clinical trials using recombinant ACE2 protein are being pursued (ClinicalTrials.gov number, NCT04287686). However, even if it might not be detrimental, recombinant ACE2 is not expected to protect from COVID-19, as circulating ACE2 upregulation correlates with the most common comorbidities in severe COVID-19. The correlation is extremely impressive and striking if we also consider the low prevalence of chronic renal disease in COVID-19 hospitalized patients (0–3%) [1,2,3,4,5,6,7,8,9] (prevalence of the disease in China ~11% [12]) which might be protected by a higher sACE2 and/or Zn^2+^ renal excretion. Indeed, in chronic kidney disease patients without a history of cardiovascular disease, there was a significant decrease in circulating ACE2 activity and zinc levels when compared with healthy control subjects [96,97]. Since proteinuria was associated with lower blood levels of sACE2 protein [153] and chronic kidney disease patients had higher urinary zinc excretion than healthy controls [97], low sACE2 activity in chronic renal diseases and protection from SARS might derive from a higher sACE2 and/or Zn^2+^ renal excretion. Indeed, sACE2 is detectable in urine of healthy subjects and urinary sACE2 protein levels are elevated in patients with chronic renal diseases and in hypertensive patients treated with the Ang II type 1 receptor blocker (ARB) olmesartan [41,154]. It can, therefore, be supposed that a basal hyperactivity of ACE2 and consequent ACE/ACE2 pathway disequilibrium in blood might predispose to the development of more severe COVID-19 symptoms that involve both arms of the RAS. In this regard, different organism predisposition to infections including SARS-CoV-2 or to pathologies associated with an increased risk to develop severe forms of SARS-CoV-2 infection might depend not only on genetic ACE2 polymorphisms and organ-specific ACE2 gene/protein expression [138,155,156,157,158,159,160,161,162,163] but also on anatomical and environmental factors. For example, ACE2 gene variants have been associated with risk for hypertension, dyslipidemia, type 2 diabetes and cardiovascular dysfunction [138,155,156,157,158,159,160,161]. Most of these variants are intronic and located in splice-site junctions or in enhancer regions while the others are placed in the 3’ UTR promoter region, suggesting their involvement in the structure/function and expression of ACE2 gene [138]. On the other hand, COVID-19 has similar risk factors of obstructive sleep apnea (OSA), suggesting a possible association between OSA and COVID-19 [164]. Indeed, high prevalence of pre-existing OSA in COVID-19 patients has been reported [165,166], indicating that OSA could be a risk factor for severe forms of Covid-19. OSA is characterised by repetitive airway collapse with apnea/hypopnea and intermittent hypoxaemia during sleep and it is associated with hypertension, cardiovascular disease, diabetes, obesity and systemic inflammation [167]. Most of these diseases are known to predispose to increased levels of circulating ACE2 and, as already mentioned, hypoxia has been shown to upregulate both arms of the RAS; therefore, OSA might predispose to the deleterious effects of SARS-CoV-2 infection. In OSA patients, upper airway collapsibility under passive conditions (critical closing pressure) is higher in males than in females due to anatomical factors, i.e., longer upper airways, as upper airway length correlates with OSA severity. Indeed, upper airways in females are less collapsible and more stable during sleep than in males [167], suggesting that an anatomical factor might contribute to and result in different gender susceptibility to COVID-19 and disease severity. Moreover, diet (e.g., zinc assumption), physical exercise and external temperature and UV radiation are all “environmental” aspects that can seasonally influence the RAS activities. Seasonal differences of the RAS activities leading, for example, to differences in blood pressure are well known. Of interest, the majority of coronaviruses cause cold-like illnesses and have a relatively low mutational frequency [168]. Similarly SARS-CoV-2 seems to mutate much slower than seasonal flu [169]. Nevertheless, like influenza virus, they are responsible for seasonal episodes of common cold in humans worldwide [168], suggesting that coronaviruses may possess an ability to evade immune control that is different from seasonal flu. For example, they might favour a relatively short-term activation/memory of immune response. Indeed, as for SARS-CoV and MERS-CoV, a short duration of immunity after SARS-CoV-2 infection has been recently suggested [170,171,172]. Seasonal cold is an annually recurring time period characterized by the prevalence of outbreaks of cold and the season occurs during the cold period of the year in each hemisphere. Altogether these observations suggest that seasonal predisposition to some virus infections (including SARS-CoV-2) might not depend only on environmental features that directly inactivate the viruses “unprotected” outside the host (e.g., higher ultraviolet rays and temperatures), but also on seasonal indirect effects produced in the organisms by environmental changes.

## 7. Monitoring ACE/ACE2 Activity in COVID-19 in Order to Determine a Rationale Use of the Specific RAS Inhibitors

There is currently no specific pharmacotherapy to combat SARS-CoV-2 infection and its pathological effects. Actually, the use of plasma from convalescent COVID-19 patients or of SARS-CoV-2-specific neutralizing antibodies has been shown to be a promising option for the treatment of critically COVID-19 patients [32,33,34,35,36,37,172,173]; however, it has some limitations that might be overcome by vaccine strategies or by the production of specific anti SARS-Co-V-2 therapeutic monoclonal antibodies. Despite the promising data that demonstrate vaccine protection against SARS-CoV-2 in nonhuman primates [174], we cannot be sure to get a vaccine against SARS-CoV-2 (see [175]). Indeed, animals immunized with inactivated SARS-CoV vaccines developed a severe (asthma-like) lung eosinophilic immunopathology when challenged with SARS-CoV, further indicating a central role of eosinophil “balanced numbers” in this pathology [176]. Vaccines might generate antibodies against viral ligand/ACE2 complex that finally blocks ACE2 activity during SARS-CoV infection and consequent downstream asthma-like events/symptoms. On the other hand, fatal outcomes in SARS-CoV infection correlated with a cytokine storm involving elevated Th2 serum cytokines (including IL-4, IL-5 and IL-10), suggesting that an increase of Th2 cytokines possibly mediated by virus-specific CD4 T cells might be crucial in the severe forms of infection [110]. In addition to cytokine profile (such as IL-10), eosinopaenia, tachycardia, normo/hypotension (although COVID-19 and hypoxia increase Ang II and many patients are “hypertensive” and/or receiving anti-hypertensive medications) and hypoxia in SARS-CoV-2 patients are compatible with downstream events stemming from both an excessive ACE2 pathway upregulation and activation of positive feedback loops (see Figure 2). ACE2 and (anti-inflammatory) IL-10 hyperproduction may ultimately trigger subsequent compensatory (and deleterious) responses, leading to both renin/ACE and pro-inflammatory cytokine upregulation. In line with this hypothesis, a longitudinal study showed that IL-6 increases late during the COVID-19 progression [3].

Altogether these data imply not only that the ACE2 is the “vehicle” of viral entry into the host cells, but also that virus-dependent and virus-independent mechanisms may sustain activation of both arms of the RAS and of the inflammatory system, finally promoting SARS-induced multi-organ injury.

Unfortunately, complete analysis of circulating levels of ACE/ACE2 proteins and activities, together with ACE and ACE2 substrates and products i.e., Ang I, Ang 1–9, Ang II, Ang (1–7) and Ang (1–5), des-Arg(9)-bradykinin and the inactive metabolite bradykinin (1–7) is lacking to address the current hypothesis. The required data could be obtained from blood sample analyses, however, it is important to consider that:

(1) Organ-specific local microenvironment might not reflect the systemic one;
(2) S1-sACE2 and SARS-CoV-2-sACE2 complexes, formed by viral-induced ACE2 shedding or by subsequent binding of sACE2 with viral particles or S1 fragments in the circulation, might not be detectable by some anti-ACE2 antibodies in ELISA. Therefore, since the complexes might prevent/mask sACE2 antibody recognition but not the enzymatic activity, sACE2 detection (and its real concentration) by ELISA in blood samples of COVID-19 patients might not be reliable;
(3) In order to evaluate circulating ACE/ACE2 activity, we can determine it by adding fluorogenic (RAS) substrates to plasma samples without (thus considering also the endogenous ACE2 inhibitor present in plasma [116]) or with adding ZnCl_2,_ which is able to induce ACE/ACE2 maximal activity [96];
(4) circulating concentrations ACE/ACE2 substrates/products likely depend on the:
  (a) level of ACE/ACE2 pathway activity and availability of substrates;
  (b) level of expression of the respective receptors (AT1R, AT2R and MasR) that bind and remove ligand products away from circulation.

For example, Ang (1–7) concentration in blood by ACE2 pathway upregulation is dependent both on the increase of the (antagonistic) Ang II (and its precursor, Ang I) and on MasR expression on surface of cells in the organs. To this regard, the detection of inactive metabolites, such as bradykinin (1–7), in COVID-19 patients and comparison with its concentration in healthy subjects could be the most indicative and reliable markers of ACE2 activity in the plasma.

Of interest, a report that described the ex vivo effect of human rACE2 on the levels of several endogenous Ang peptides in plasma samples, thus mimicking an acute increase of systemic ACE2 activity in plasma [115] showed a strong reduction of Ang II (as expected), a moderate reduction of Ang I and a significant (more than five-fold) increase of Ang (1–9), Ang (1–7) and (ACE-produced) Ang (1–5) [115]. Therefore, Ang (1–5) may also be an interesting surrogate marker of ACE-ACE2 combined activity in COVID-19 patients [115]. 

At the present time, it has been reported that plasma levels of Ang II in SARS-CoV-2 infected patients are markedly elevated as compared with healthy subjects (interquartile range between 260–360 pg/mL and 65–120 pg/mL, respectively) and linearly associated to viral load and lung injury [177]; unfortunately, nobody has tested the levels of Ang (1–7), Ang (1–9), Ang (1–5) and bradykinin (1–7), some of which might be at high levels as well, thus justifying both normo/hypotension and eosinopaenia in COVID-19 patients. More information on the RAS peptides in plasma of Acute Respiratory distress syndrome, a COVID-19-like disease, (and pulmonary arterial hypertension) is instead available (see Box 6).

Box 6 **Acute Respiratory distress syndrome and hyperactivation of the RAS pathways.**

The main clinical feature of ARDS patients is the progressive deterioration of lung function leading to progressive hypoxemia. ARDS can be induced by different causes such as ZnCl_2_ aspiration, sepsis, trauma, acute pancreatitis, or pneumonias following virus infections such as SARS-CoVs or human influenza virus. ACE insertion/deletion polymorphisms correlated with severity of ARDS in humans, suggesting that the RAS could have a role in ARDS [44]. In animal models, ACE2 protected from acute lung by inhibiting Ang II/AT1R activity [15], for this reason the use of recombinant ACE2 in ARDS has been proposed and pursued [134]. Clinical trials with recombinant human ACE2 started in healthy volunteers in 2009 (ClinicalTrials.gov number, NCT00886353 [178]), then it was tested in ARDS (ClinicalTrials.gov number, NCT01597635 [134]), PAH (ClinicalTrials.gov number, NCT01884051 [108]) and finally in COVID-19 patients (ClinicalTrials.gov number, NCT04287686). Despite its safe use, no conclusive evidence in support of its use in these diseases was reported.

Of interest, in the plasma samples from ARDS patients, levels of Ang I have been shown to be significantly increased in non-survivors (interquartile range 1990–16950 pg/mL) compared to survivors (interquartile range 730–5660 pg/mL) [92], suggesting a marked increase of renin activity in non-surviving ARDS patients. Unfortunately, no comparisons of the two ARDS groups with healthy subjects (interquartile range 35–66 pg/mL, markedly lower than both patient groups [179]) were reported; this is important to consider since the plasma concentrations of the RAS peptides may significantly differ depending on method of detection (ELISA versus vs. mass spectrometry) and, in particular, on protocol of determination (endogenous versus equilibrium Ang peptide levels [179]). Endogenous RAS peptide concentrations can be evaluated collecting and stabilizing blood samples with a RAS enzyme inhibitor cocktail. Differently, heparinized plasma samples can be incubated at 37 °C to induce ex vivo equilibrium, which unveils the presence of renin, ACE and/or ACE2 activity in the plasma (equilibrium Ang peptide levels) [179]. This last protocol usually gives significantly higher levels of the RAS peptides [179]. For example, in healthy subjects, the normal ranges (interquartile ranges) of endogenous RAS peptides, Ang I, Ang II, Ang (1–7), Ang (1–9) and Ang (1–5) were 12–24 pg/mL, 1–12 pg/mL, <2 pg/mL, <4 pg/mL and < 1 pg/mL, respectively, differently the normal range after ex vivo equilibrium of the same peptides were 35–66 pg/mL, 110–200 pg/mL, < 2 pg/mL, < 4 pg/mL and < 1 pg/mL, respectively [179], indicating the presence of renin and ACE (but no ACE2) activity (as highlighted by Ang peptide increases upon ex vivo equilibrium). Similarly, another report that evaluated Ang II and Ang (1–7) in healthy males showed normal endogenous ranges ~ 14.5 pg/mL and 0.9 pg/mL, respectively, and an Ang (1–7)/Ang II ratio ~ 0.06 ratio [180]. Moreover, Ang (1–7), Ang I, Ang (1–9) and Ang (1–7)/Ang II ratio were significantly higher in ACEI-treated hypertensive patients compared to healthy subjects [180], suggesting that inhibition of ACE predisposes to upregulation of the ACE2/Ang (1–7)/MasR pathway. Notably, two different reports that evaluated the RAS peptides in ARDS patients using the similar method of detection (high performance liquid chromatography plus mass spectrometry) showed significantly different results. One report showed that Ang II ranged (interquartile range) between 120–630 pg/mL (non-survivors) and 70–2220 pg/mL (survivors) [92]. A second report showed significantly lower values of Ang II, (interquartile range) between 40–150 ng/mL (non-survivors with placebo) and 5–20 pg/mL (survivors with placebo) (see Supplementary Data [134]), suggesting that they used different protocols of the RAS peptide determination. Nevertheless, both Ang (1–7) and Ang (1–7)/Ang II ratios found in ARDS patients of both reports [92,134] were significantly higher than healthy subjects. One report showed that Ang (1–7) ranged (interquartile range) between 2.5–10 pg/mL (see Figure 2B of [134]), Ang II between 5–20 pg/mL (see Figure 2 of [134]) and Ang (1–7)/Ang II ratio ~ 0.5. Moreover, in the report in which the elevated concentrations of Ang I and Ang II indicate an ex vivo equilibrium of the RAS peptides, the increase was even more marked. In that report, Ang (1–7) (< 1pg/mL in healthy subjects) ranged between 80–1070 pg/mL and Ang (1–7)/Ang II ratio (~ 0.06 in healthy subjects) ranged between 0.24–1.82 [92], suggesting that ACE2 activity (and ACE2 presence in plasma samples) was significantly increased in ARDS patients as compared to healthy subjects. In line with this hypothesis, there was the determination of Ang (1–9), whose production from Ang I exclusively depends on ACE2 (which also compete with ACE for the same substrate). In normal healthy subjects, it was reproducibly below the limit of quantification [179,180], differently, both in ex vivo treatments of plasma samples with human rACE2 [115,181] and in ARDS patients, it was markedly increased (Ang (1–9) ranged between 100–3080 pg/mL) [92], suggesting both that the dominating activity of ACE in plasma of healthy subjects is overcome by that of ACE2 in ARDS patients. Therefore, Ang (1–9) may represent a good surrogate marker for ACE2 activity. Similarly to Ang (1–9), there was the determination of Ang (1–5), whose production depend on both ACE2 [that produces Ang (1–7) from Ang II] and ACE [that produces Ang II from I and Ang (1–5) from Ang (1–7)]. Ang (1–5) was undetectable in healthy subjects [178,179]; however, it was significantly increased in ARDS patients (interquartile range between 50–730 pg/mL [92]), suggesting that both ACE2 and ACE activity were likely upregulated as compared with healthy subjects. The activation for both arms of the RAS needs of the contemporary activation renin, the rate-determining enzyme of the RAS. Indeed, analysis of plasma samples (from a healthy subjects), treated ex vivo with recombinant renin, showed an increase of Ang I, Ang II, Ang (1–7) and Ang (1–5), but not Ang (1–9) [115,179,181], further suggesting both the ACE2 specific activity in producing Ang (1–9) and an activation of both arms of the RAS in ARDS patients. Finally, in vivo or ex vivo administration of human rACE2 in plasma samples produced an increase not only of Ang (1–9) but also of Ang (1–5) and Ang (1–7) in both healthy subjects [178] and ARDS patients [134], indicating that all three Ang peptides might represent surrogate markers of ACE2 activity. Of interest, the mean arterial pressure of ARDS patients was markedly low and significantly lower in non–survivors (mean value 65 mmHg and more likely need vasopressor support) compared to survivors (mean value 71 mmHg) [92], which may likely be a consequence of the above described ACE2 upregulation. Interestingly, similar features were also detected in PAH and coronary atherosclerosis. In particular, a significant increase of both Ang II and Ang (1–7) peptides was observed in plasma of these patients [108,182] and plasma levels of TNF-α were significantly elevated in the critical coronary artery disease [182]. Moreover, despite a significant increase of Ang II/Ang (1–7) ratio (that suggested a therapeutic use of recombinant hACE2 in PAH patients) systolic and diastolic blood pressures were in the range of normality [108,182]. Altogether these observations suggest that Ang peptide ratios are not reliable markers for disease status in these pathologies; rather, increased plasma amounts of each RAS peptide should be careful considered.

## 8. Hypothesizing Pharmacological Treatments for COVID-19

Based on the above described hypothesis, inhibition of ACE2 pathway might be beneficial for COVID-19 patients. Indeed, the clinical picture as a whole is consistent with an ACE2 gain of function (possibly due to both circulating active forms of S1-sACE2 complexes and local forms of SARS-CoV-2-sACE2 complexes) rather than an ACE2 loss of function, as initially supposed. Therefore, inhibition of ACE2/Ang (1–7)/MasR axis or other ACE2 pathways to restore ACE/ACE2 balance might be needed, at least in the first phases of the disease when hypoxia is not yet induced. Different strategies could be pursued through ACE2 pathway inhibitors and/or MasR antagonists and/or renin inhibitors and/or metal chelators.

Several different molecules have been designed to specifically inhibit human ACE2 enzyme (both membrane bound and soluble forms) or human MasR signal transduction, but only a few have been studied in vivo. Some ACE2 pathway inhibitors have been widely used in mouse/rat models, as control of human and mouse ACE2 activity or in mouse models of colitis. Thanks to mouse/rat in vivo experiments and clinical trial results in human participants it has been possible to infer toxicity/efficacy for some human ACE2 inhibitory molecules (MLN-4760/C16/GL1001/ORE1001, Dx600 and A779) that may be exploited to face this exceptionally dramatic situation. Unfortunately, to my knowledge, only one (MLN-4760/C16/GL1001/ORE1001) of the ACE2-specific inhibitors has been tested in vivo in humans in a Phase I clinical trial long time ago (http://oreholdings.com/wp-content/uploads/2013/06/09.02.09-S-4-A.pdf). 

Here, I have focused my attention on inhibitors of human ACE2 pathway that were consistently administered in vivo. Making use of published reports in which human/rodent ACE2 pathway inhibitors were administered in vivo, I have hypothesized a possible therapeutic pharmacological intervention through an inhibition strategy of the RAS pathways for COVID-19 in patients experiencing both mild and critical, advanced and untreatable stages of the disease (the most problematic cases to manage).

### 8.1. Inhibition of ACE2: MLN-4760

Briefly, one of the best candidates to treat COVID-19 patients, but not to prevent viral entry, is the small synthetic molecule MLN-4760 (specific ACE2 inhibitor, also known as C16, GL1001 or ORE1001, [113]) for the following reasons:

(1) It has been shown to bind/inhibit ACE2 enzymatic activity even at low/acidic pH (pH 6.5, [115]) typical of hypercapnia (as it might occur in lungs of COVID-19 patients) when human ACE2 activity is maximal [79]; nevertheless, it retains its inhibitory effects on soluble ACE2 bound to spike proteins [24], indicating that it is able to bind and inhibit ACE2 activity regardless ACE2 binding to SARS-CoV-2 particles or to S1 fragments.
(2) No significant adverse effects were described upon its chronic administration neither alone nor in combination with ACE2 activators (while inhibiting their activating effects) nor after inducing functional impairment of ACE2 activity in rodent experiments in vivo [52,117,118,122,183,184] nor in a clinical Phase I trial in humans (http://oreholdings.com/wp-content/uploads/2013/06/09.10.09-425.pdf);
(3) Its administration by different route is well described in rodents and humans. In particular:
  (a) Chronic administration (about 4 weeks) of C-16/DLM-4760 in combination with ACE2 activating treatments was performed by daily intraperitoneal injection at a dose of 25mg/kg in distilled water (as a solution of 42 mg/mL) or 0.9% sterile saline (as a solution of 84 mg/mL using a 0.5-mL insulin syringe) freshly prepared [52,183,184].
  (b) Alternatively, chronic administration (about 8 days) of GL1001/DLM-4760 disodium salt in combination with an ACE2 activating treatment was performed by subcutaneous injection (5mL/kg) containing up to a dose of 300 mg/kg, twice a day, formulated in a vehicle solution [15% 2-hydroxypropyl-β-cyclodextrin (HPBDC)/85% H_2_O] [122]. Subchronic doses of GL1001 indicate no adverse effects up to 1,000 mg/kg (see [122]).
  (c) In humans ORE1001/GL1001/MLN-4760 was already proposed and tested in clinical trials. Its pharmaceutical indication was for digestive tract inflammations (Inflammatory bowel disease, gastritis and colitis) that are correlated with overexpression of ACE2. In a Phase I clinical testing up 14 days dosing, ORE1001 was well tolerated. Subjects received drug (dosing up to 2100 mg) with no side adverse effects reported. In particular, 47 subjects received single-dose from 2.1 to 2100 mg and 24 subjects received 14 day multiple doses from 50 mg to 1800 mg. All doses were well tolerated, with no significant side effects including blood pressure. Pharmacokinetics of orally administered capsules was consistent with once-daily dosing. (http://oreholdings.com/wp-content/uploads/2013/06/09.10.09-425.pdf). 300 mg (active drug) oral capsules were used in a Phase Ib/IIa clinical trial that was, however, abandoned. (https://clinicaltrials.gov/ct2/show/NCT01039597).
  (d) Finally, MLN-4760 was also administered (2.5 mg/kg per day) by nasal inhalation for 2–3 days in lung-infected mice by *Pseudomonas* bacteria [185]. Interestingly, the report underscores the role played by local concentration of molecules (ACE2) in modulating lung inflammation and disease. For these reasons, in diseases involving respiratory tract, like SARS, inhalation treatment is preferable, even for the lower concentration (and hopefully lower toxicity) of MLN-4760 needed for this route of treatment administration.

Different routes of MLN-4760 treatment administration can be pursued depending on the hospital condition/expertise. In this exceptionally critical situation, it could be delivered to critical untreatable patients as a controlled “compassionate use”, in particular by inhalation. However, when, under hypoxic conditions, both arms of the RAS are upregulated, it might have a limited action and, by shifting the balance of ACE/ACE2 ratio in favour of ACE, might be even dangerous. Instead, specific inhibition of ACE2 enzymatic activity might effectively work in preventing the establishment of positive feedback loops in COVID-19 patients who are suffering from mild symptoms of the disease. MLN-4760 is sold by different companies, that, in case it works, could be encouraged to manufacture the molecule, actively contributing to face this global threat. On the other hand, the drug could be also synthesized in University chemistry labs because, to my knowledge, is no longer under patent restriction. MLN-4760, whose clinical development was abandoned after phase I trials (clinical name, ORE1001), is actually an interesting compound that could be useful for all patients in which a specific ACE2 upregulation is proven and associated to different (heart/lung/liver/intestine/kidney/immune system/blood/coagulation, etc.) pathological conditions related to ACE2 pathway downstream events, as it seems to occur in COVID-19 patients. Although in blood of normal subjects sACE2 activity is undetectable because its catalytic activity in human plasma is masked by an endogenous inhibitor [116] and chronic ACE2 activation may be deleterious, some patients might need of sACE2 activity to better face an acute pathological condition, therefore a careful monitoring of clinical parameters should be performed during patient treatment.

### 8.2. Inhibition of Renin Activity: Aliskiren

An alternative approach is to inhibit the RAS pathway at its origin by inhibiting renin enzymatic activity. Regarding renin inhibitors, aliskiren is the sole compound in this class of drugs that was approved by the US Food and Drug Administration in March 2007 and commercialized for the management of hypertension [186]. Aliskiren may be used alone or in combination with other drugs to face COVID-19, in particular the severe forms of SARS-CoV-2 infection. Indeed, in patients with severe symptoms of COVID-19 hypoxia might have upregulated both arms of the RAS. Therefore, aliskiren might be a useful “tool” to reduce production of Ang I, the necessary fuel for both ACE and ACE2 hyperactivity and their detrimental effects. Aliskiren treatment in rats has been shown to upregulate both AT1R and MasR expression probably as consequence of a compensation mechanism when both Ang II and Ang (1–7) ligands are reduced by renin inhibition [187]. Of particular interest in the context of SARS-CoV-2 infection, the treatment has been shown to reduce expression of both AT2R and ACE2, thus possibly performing a multiple action in inhibiting both arms of the RAS [187]. Similarly, cation chelating agents such as CaNa_2_EDTA or nicotianamine might also work, although at different levels.

### 8.3. Chelating Agents: CaNa_2_EDTA

As already mentioned, chelating agents, by limiting zinc cellular availability, might influence hyperactivity, synthesis and conformation of both ACE2 and ACE, which may impair not only ACE2 (and ACE) enzymatic activity but also its availability on cell surface for viral entry. For these reasons, chelating agents might be effective to counter SARS-CoV-2 infection, and in particular when (e.g., induced by hypoxia) both arms of the RAS are likely upregulated. Differently from renin inhibitors, more chelating agents are commercially available and both classes of drugs might be administered, alone or in combination with other therapies for COVID-19. Of note, these classes of drugs might be beneficial not only for SARS-CoV-2 infection but also for other disease in which both arms of the RAS (and of the inflammatory system) are upregulated, as for example ARDS, PAH and other pathologies associated to chronic hypoxia and cardiac failure. Indeed, both Ang-II and Ang-(1–7) upregulation and a cytokine storm are common aspects in patients not only with ARDS and PAH but also with critical coronary artery disease [182], suggesting an upregulation of both arms of the RAS and of the inflammatory system in several different dysfunctions. Notably, a number of clinical trials have indicated the utility of CaNa_2_EDTA in coronary heart disease. It was believed that chelation therapy might alter plaque morphology and volume, or improve endothelial function [123]. However, the evidence was not convincing [123]. Future clinical trials on selected patients with both ACE and ACE2 upregulation might be more convincing. It is clear that similar pathways of activation can lead to different outcomes depending on individual predisposition/background. Therefore, future efforts to understand the genetic, morpho-physiological, environmental and lifestyle factors that individually affect the predisposition to a specific disease with a precision medicine must be pursed. 

To avoid disrupting extracellular calcium levels, CaNa_2_EDTA formulation is typically employed as an antidote for lead poisoning or as an extracellular zinc chelator [123]. CaNa_2_EDTA is poorly absorbed in the gastrointestinal tract therefore can be preferentially administered by parenteral route or by inhalation. Nevertheless, oral tablets (e.g., 250–500 mg/tablet) or suppositories of CaNa_2_EDTA might also work in reducing plasma zinc levels by inhibiting intestinal zinc absorption (the only physiological way of zinc uptake by the organism) [188,189]. In this regard, eight men showing elevated lead concentrations were given a seven-day course of CaNa_2_EDTA, 4 g/day in divided doses. Oral CaNa_2_EDTA caused a rise in lead excretion within few hours, suggesting that orally administered drug was partially absorbed [188]. Importantly, the trial in the eight lead workers demonstrated that oral administration of CaNa_2_EDTA was safe and produced no side-effects [188]. However, the FDA-approved method for delivery of CaNa_2_EDTA in treatment of lead poisoning is intravenously or intramuscularly and the recommended dose for asymptomatic adults and pediatric patients with elevated blood lead levels is 1000 g/m^2^/day (~0,05 g/kg/day) [190]. CaNa_2_EDTA was usually administered for 5–10 days followed by an interval of at least 5–10 days before a second administration. Unfortunately, intravenous administration is very painful. Administration of CaNa_2_EDTA via nebulizer has also been pursued for inhalation therapy in pulmonary metal poisonings to protect lung tissue and reduce its systemic uptaken [123]; moreover, nebulized solutions containing EDTA have been proven safe, not inducing adverse effects [191]. For the sake of completeness, the unconventional methods for administering chelating agents such as rectal or inhalatory administration are not FDA approved methods for delivery and they have not been studied in detail [192]. CaNa_2_EDTA diffuses mainly in the extracellular fluids, where it is not significantly metabolized, and it is excreted rapidly by glomerular filtration. The chelator has a half-life of 1.4 to 3 h in adults and is completely excreted within 24 h [123]. For all of the above reasons, CaNa_2_EDTA might be employed in therapy of COVID-19. However, before beginning any therapy with medication, particularly in the case of new diseases (such as COVID-19) or new treatments, a careful patient selection for these new treatments is essential to prevent unnecessary toxicity (“first do not harm”).

### 8.4. Safety and Efficacy Concerns

Unfortunately, diseases of different aetiologies may present similar final dysfunction/alterations (e.g., cardiac dysfunction/lung alterations/thrombosis are induced by excessive activation of either AT1R or MasR/AT2R pathways, see Figure 1), while diseases with same aetiologies may present different final dysfunction/alterations depending on patients (COVID-19 is a clear example). This confounding aspect of diseases can lead to erroneous interpretations, and only defining the correct and early molecular origin of diseases is possible to reach a successful outcome if a therapy is available.

In order to decide who to treat, and when to start treatment, it is important to define the entity of dysfunctions, probability of treatment success and probability to develop adverse effects. To promptly intervene in order to prevent severe forms of COVID-19, we need early markers of disease progression. To this regard, if plasma (free) Zn^2+^ (or total zinc/albumin molar ratio), albumin, IL-10, Ang (1–9), Ang (1–7), Ang (1–5) and/or bradykinin (1–7) will be proven to be reliable surrogate markers for ACE and/or ACE2 enzymatic activity in vivo, they could be exploited, together with circulating ACE and ACE2 activity evaluation, in order to decide who and when to start treating COVID-19 patients. Alternatively, eosinopaenia and hypotension may be also exploited as signs of disease progression. In any case, since the conditions of some patients can be critical, it is highly recommended to administer the minimal effective dose in order to reduce possible side effects, starting with low and increasing doses, while at the same time monitoring patient status and early signs of disease progression (e.g., blood pressure, eosinophil counts, molecular concentrations of the RAS peptides and other markers of inflammation). The aim of the project was not necessarily to stop viral entry (the epithelial cells take already care of it shedding ACE2) but to block positive feedback loops that follow the cellular response to SARS-CoV-2 infection, which are likely the main cause of the severe symptoms associated with COVID-19, doing so, the disease might hopefully become like a simple flu, a spontaneously eradicable disease.

At the moment, I am alone in proposing these new and non-conventional therapeutic approaches that may or may not work on COVID-19. In doing so, I have not only described a rationale/strategy to counter COVID-19, but also provided practical information to optimize the RAS inhibitors based on known experimental data. Before any in vivo drug administration (e.g., ACE2 inhibitor), it has to be checked the biological activity (e.g., ACE2) that is affected by the drug, considering the possible side effects. However, this is not always the case and, in some cases, patients are treated without a careful evaluation of the biological parameter that is modulated by the administered drug. In this regard, I have tried to address all aspects of the RAS inhibition in detail because my main concern is that the use of these approaches in a wrong way may lead to a failure or even to deleterious effects. That is why I have meticulously described all the steps before possible administration and provided the guideline suggestions and specific recommendations in order to increase the probability of success and to minimize the deleterious effects. The readers are, however, expected and encouraged to debate and make their own optimized guidelines.
